# Reconfigurable terahertz metamaterials: From fundamental principles to advanced 6G applications

**DOI:** 10.1016/j.isci.2022.103799

**Published:** 2022-01-21

**Authors:** Cheng Xu, Zhihao Ren, Jingxuan Wei, Chengkuo Lee

**Affiliations:** 1 Department of Electrical & Computer Engineering, National University of Singapore, 4 Engineering Drive 3, Singapore 117576, Singapore; 2 Center for Intelligent Sensors and MEMS (CISM), National University of Singapore, 5 Engineering Drive 1, Singapore 117608, Singapore; 3 National University of Singapore Suzhou Research Institute (NUSRI), Suzhou Industrial Park, Suzhou 215123, China; 4 NUS Graduate School for Integrative Science and Engineering, National University of Singapore, Singapore 117456, Singapore

**Keywords:** Radiation physics, Broadband communication, Metamaterials

## Abstract

Terahertz (THz) electromagnetic spectrum ranging from 0.1THz to 10THz has become critical for sixth generation (6G) applications, such as high-speed communication, fingerprint chemical sensing, non-destructive biosensing, and bioimaging. However, the limited response of naturally existing materials THz waves has induced a gap in the electromagnetic spectrum, where a lack of THz functional devices using natural materials has occurred in this gap. Metamaterials, artificially composed structures that can engineer the electromagnetic properties to manipulate the waves, have enabled the development of many THz devices, known as “metadevices”. Besides, the tunability of THz metadevices can be achieved by tunable structures using microelectromechanical system (MEMS) technologies, as well as tunable materials including phase change materials (PCMs), electro-optical materials (EOMs), and thermo-optical materials (TOMs). Leveraging various tuning mechanisms together with metamaterials, tremendous research works have demonstrated reconfigurable functional THz devices, playing an important role to fill the THz gap toward the 6G applications. This review introduces reconfigurable metadevices from fundamental principles of metamaterial resonant system to the design mechanisms of functional THz metamaterial devices and their related applications. Moreover, we provide perspectives on the future development of THz photonic devices for state-of-the-art applications.

## Graphical abstract

**Figure:**
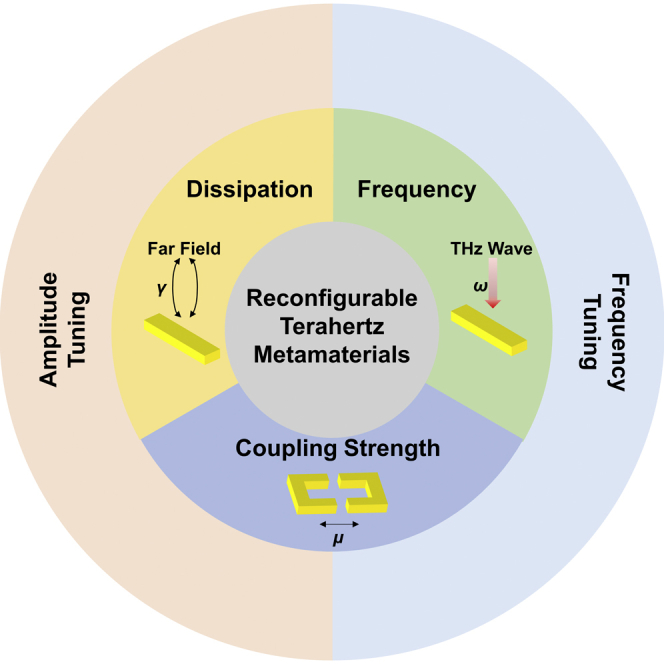

## Introduction

Terahertz (THz) electromagnetic waves have a frequency range from 0.1THz to 10THz, which lies between microwave frequencies and optical wave frequencies. Benefitting from the advantages of both neighboring spectra regions, the THz band enables innovative and similar applications, such as communication and sensing (Siegel, 2002; Ferguson and Zhang, 2002; Fitch and Osiander, 2004; Tonouchi, 2007; Sizov and Rogalski, 2010; Yang and Lin, 2020). On the one hand, while the millimeter-wave band dominates in the 5G communication systems over the past few years, the THz band is critical to the development of 6G communication considering its abundant bandwidth, lower latency, and enhanced data transfer rate from Gbps to Tbps level (Rappaport et al., 2019; Elayan et al., 2020). On the other hand, compared with the infrared spectroscopic sensors (Dong et al., 2018; Qiao et al., 2019; Chang et al., 2020a, 2020b), THz sensors have also attracted a lot of attention (Yang et al., 2016; Gupta et al., 2017) not only due to observable intramolecular and intermolecular vibrational modes of many chemicals and biological macromolecules in this region but also because of the non-destructive, non-ionization, and non-invasive properties, when compared with other frequencies. However, known as the “THz gap”, owing to the limitation of electronic and photonic devices in this region, efficient conversion between electromagnetic waves and electrical power becomes difficult, lacking relevant functional devices to bridge this technology gap.

Metamaterials are artificially composed subwavelength structures. With their artificially engineered electrical properties, exotic physical phenomena have been observed in metamaterials, such as negative refractive index (Pendry, 2000; Shelby et al., 2001), metalens (Chen et al., 2017; Wang et al., 2018b; Wang et al., 2019b; Ren et al., 2021a, 2021b), slow light effect (Choi et al., 2011; Ma et al., 2018a; Ma et al., 2018b; Sun et al., 2020) (Ma et al., 2020a, 2020b), and perfect absorption (Hasan et al., 2017; Hasan and Lee, 2018; Kang et al., 2019). Known as “unit cell” or “meta-atom”, the unit structures can vary widely in the geometric parameters. Therefore, metamaterials-based functional electromagnetic structures with scalable spectral, subwavelength shapes are widely demonstrated in the THz region (Chen et al., 2006; Chen et al., 2008; Chen et al., 2009).

**Figure 1:**
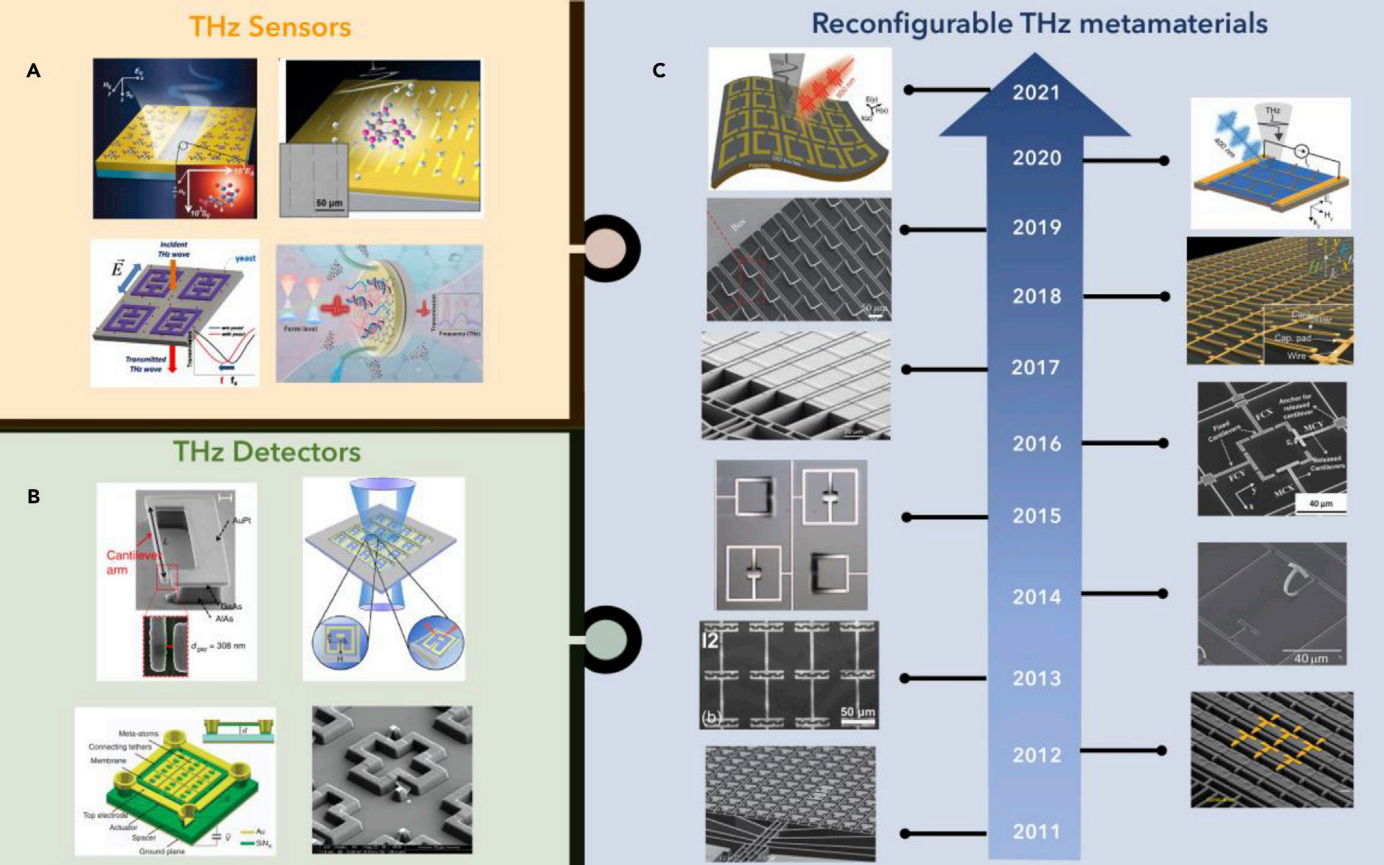
A roadmap of the development of reconfigurable THz metamaterials in the past 10 years, and metamaterials-enabled THz functional devices Reprinted from ref (Tao et al., 2011; Grant et al., 2013; Liu et al., 2017; Belacel et al., 2017; Park et al., 2013; Lee et al., 2015; Tenggara et al., 2017; Zhou et al., 2021c; Zhu et al., 2011; Zhu et al., 2012; Li et al., 2013; Ma et al., 2014; Pitchappa et al., 2015a, 2016c; Zhang et al., 2017; Zhao et al., 2018; Cong et al., 2019; Pitchappa et al., 2020, 2021b) with permission, Copyright@2011 Optical Society of America, Copyright@2013 Wiley-VCH, Copyright@2017 Spring Nature, Copyright@2017 Spring Nature, Copyright@2013 American Chemical Society, Copyright@2015 Spring Nature, Copyright@2017 IOP Publishing, Copyright@2021 Elsevier, Copyright@2011 Wiley-VCH, Copyright@2012 Spring Nature, Copyright@2013 AIP Publishing, Copyright@2014 Spring Nature, Copyright@2015 Optical Society of America, Copyright@2016 Wiley-VCH, Copyright@2017 Spring Nature, Copyright@2018 Optical Society of America, Copyright@2019 AAAS, Copyright@2020 Wiley-VCH, Copyright@2021 Wiley-VCH.

Recent advanced technologies hold promises for the dynamic modulation of electromagnetic waves. To implement the active control of electromagnetic waves, many functional materials have been demonstrated in the past decade, such as phase change materials (Rensberg et al., 2016; Qu et al., 2017; Shaltout et al., 2019), photoactive materials (Padilla et al., 2006; Chen et al., 2008; Manjappa et al., 2017), ferroelectric materials (Kang et al., 2008; Zhao et al., 2009; Qi et al., 2009), and liquid crystals (Reuter et al., 2013; Shrekenhamer et al., 2013; Savo et al., 2014). The tunable states of the materials enable the changes in optical properties, such as the refractive index (n) and extinction coefficient (k), which can be activated electrically, optically, thermally, or dynamically. Among these tuning mechanisms, two solutions have raised the attention and been reported frequently, microelectromechanical systems (MEMS) technology and tunable two-dimensional (2D) materials. On the one hand, the geometric-dependent metamaterial resonators could be effectively influenced by changing the configuration of unit cells, which can be enabled by MEMS actuators to a broadband frequency ranging from THz to visible light (Lin and Lee, 2014; Dong et al., 2020a; Ma et al., 2020b; Ren et al., 2020; Dong et al., 2020b; Lin and Xu, 2020; Pitchappa et al., 2021a; Zhou et al., 2021a; Wang et al., 2021). Moreover, the integration of MEMS actuators and metamaterials are compatible with the current complementary metal-oxide-semiconductor (CMOS) fabrication platform. On the other hand, 2D materials such as graphene have a low carrier density of states, which enables the tuning of Fermi level by applying a gate voltage (Tassin et al., 2013; Kakenov et al., 2018; Liu et al., 2018). Thereby, the conductivity of graphene, as well as the resonance frequency will be changed, making it possible for reconfigurable metamaterials tuning devices. Combining the tunable metamaterials with THz photonics, functional THz devices could be effectively utilized to fill the THz gap, as shown in Figure 1. Metamaterial-patterned THz sensors enable the enhancement of near-field intensity, improving the interaction between THz light and molecules for advanced sensing applications, as shown in Figure 1A (Park et al., 2013; Lee et al., 2015; Tenggara et al., 2017; Zhou et al., 2021c). In addition to that, for communication systems, metamaterials-enabled THz detectors can absorb THz electromagnetic waves with selective frequencies and strong resonance strengths, as shown in Figure 1B (Tao et al., 2011; Grant et al., 2013; Liu et al., 2017; Belacel et al., 2017). Nevertheless, because metamaterials are usually lithographically determined and cannot be further modified once the fabrication process is complete, the fixed working range will limit the functionality of THz metadevices. Therefore, reconfigurable metadevices are more competitive, especially when dealing with complicated systems, where programmable design can benefit signal processing algorithms for a large amount of data, providing opportunities for the assistance of artificial intelligence for healthcare, environmental monitoring, reconfigurable intelligence surface for wireless communication systems, and Internet of Things applications.

A roadmap showing the development of metamaterial-enabled reconfigurable devices in the past 10 years is shown in Figure 1C. In 2011, Zhu et al. demonstrated an in-plane electrostatic comb drive actuator, manipulating one split magnetic metamaterial resonator (Zhu et al., 2011). Later in 2012, the same group demonstrated similar comb drive structures, but with anisotropic response to the incident polarization state of THz waves, adding more functionalities to THz devices (Zhu et al., 2012). In 2013, Li et al. proposed a stretchable THz metamaterial, which leveraged the tuned gap difference between each resonator. These flexible substrate devices fabricated in the THz region enabled potential applications in biocompatible strain sensing (Li et al., 2013). One year later, Ma et al. demonstrated out-of-plane cantilever metamaterials and proposed for frequency modulation application in the THz region (Ma et al., 2014). Moving forward, the same group demonstrated a metadevice for digital state control leveraging both magnetic resonators and electrical resonators (Pitchappa et al., 2015a). One year later, owing to the significant information brought by the polarization states and its potential for polarization converter, and waveplate applications, Pitchappa et al. proposed a polarization-dependent THz MEMS metamaterial design with multiple states control (Pitchappa et al., 2016c). Later in 2017, Zhang et al. demonstrated a THz metasurface with reconfigurable polarization conversion and rotation, which is based on in-plane deformable designs (Zhang et al., 2017). Then in 2018, Zhao et al. proposed a tunable THz waveplate leveraging out of plane cantilever design. Furthermore, the polarization states were also quantized by applying different voltages, realizing transformation between multiple states (Zhao et al., 2018). One year later, Cong et al. demonstrated THz reconfigurable circular polarization control utilizing asymmetric metasurface design, indicating more complicated polarization manipulation for THz metadevices (Cong et al., 2019). In 2020, cantilever-based metamaterial-enabled hybrid modulation of amplitude and frequency in the THz region was first demonstrated by Pitchappa et al., where electrical and optical control was decoupled for independent manipulation of THz information (Pitchappa et al., 2020). Owing to the increasing demand for ultra-high-speed applications for 6G communication, functional tunable devices with high response rates have arisen significant attention recently. In this year, Pitchappa et al. proposed a flexible metadevice integrated with phase change materials, where picosecond-level time delay was demonstrated for the reconfiguration process (Pitchappa et al., 2021b).

This review will be organized into five major parts. In Tuning mechanisms of THz reconfigurable metamaterials, the multiple tuning mechanism of reconfigurable metamaterials will be introduced and discussed. Then in THz reconfigurable metamaterials with single resonance and THz reconfigurable metamaterials with multiple resonances, we will divide the metadevices into single resonant systems and multiple resonant systems, explaining their principles and relative applications, respectively. In Application of tunable THz metamaterial devices, several applications including the amplitude and phase modulation process will be conducted. Furthermore, a summary of the tunable metamaterials with their functional metadevices, as well as a perspective of future THz photonics devices will be concluded in Conclusion and Outlook.

## Tuning mechanisms of THz reconfigurable metamaterials

### Microelectromechanical system tuning mechanisms

**Figure 2:**
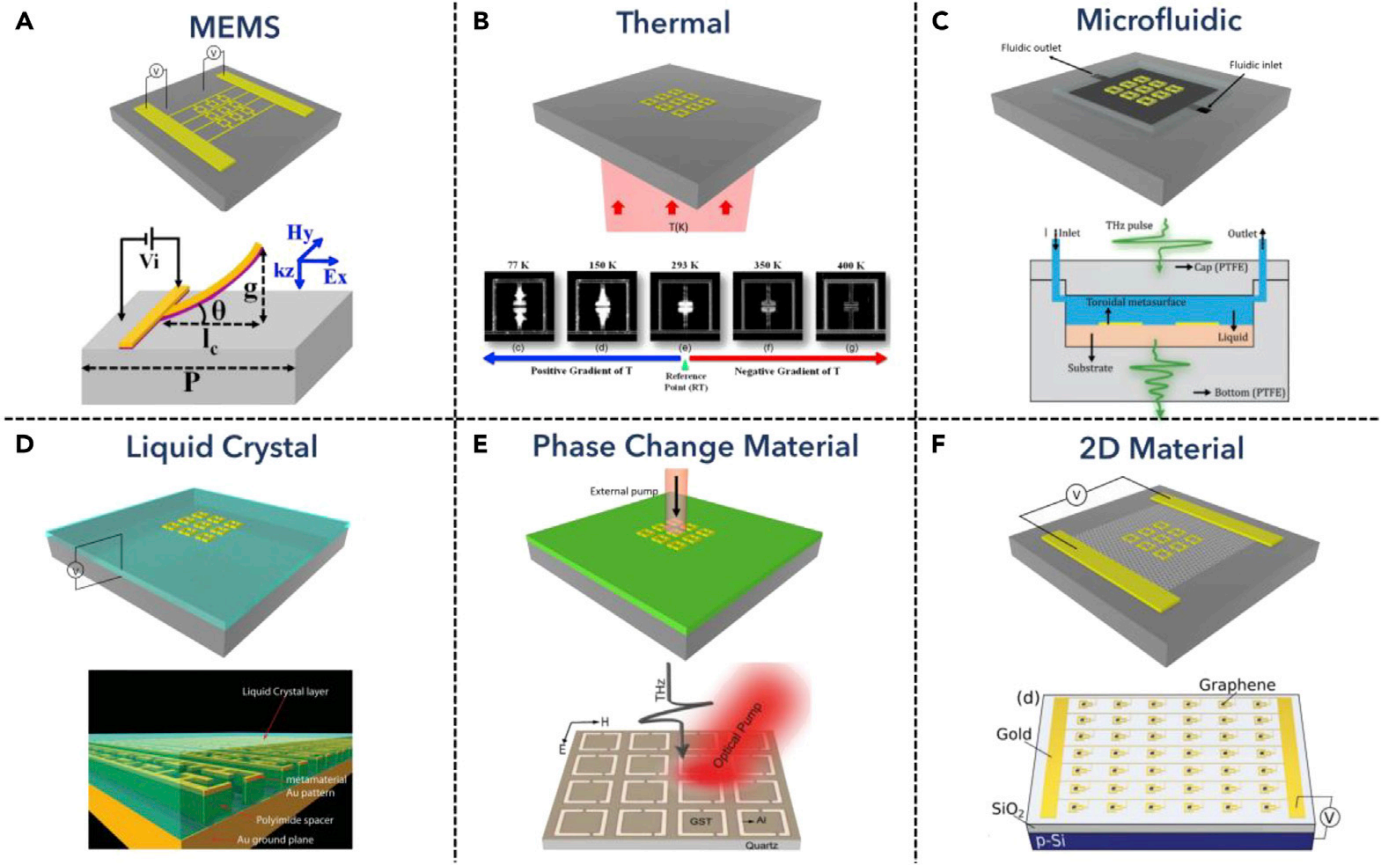
Working mechanisms of tunable metadevices Reprinted from ref (Pitchappa et al., 2015c; Pitchappa et al., 2017; Xu et al., 2021a; Savo et al., 2014; Pitchappa et al., 2019; Kindness et al., 2018) with permission, Copyright@2015 IEEE, Copyright@2017, AIP Publishing, Copyright@2021, Wiley-VCH, Publishing, Copyright@2014 Wiley-VCH, Copyright@2019 Wiley-VCH, Copyright@2018 Wiley-VCH. (A) A schematic showing MEMS actuator-based tunable THz metamaterials, and an example showing cantilever deformable design with external electrical power applied. Reprinted from (Pitchappa et al., 2015c) with permission, Copyright@2015 IEEE. (B) A schematic showing thermal elastic-based tunable THz metamaterials, and an example showing thermal-dependent metamaterial resonator design with temperature varied from 77 K to 400 K. Reprinted from (Pitchappa et al., 2017) with permission, Copyright@2017 AIP Publishing. (C) A schematic showing microfluidic-based tunable THz metamaterials, and an example showing the building blocks of microfluidic chamber design. Reprinted from ref (Xu et al., 2021a) with permission, Copyright@2021 Wiley-VCH. (D) A schematic showing liquid crystal-based tunable THz metamaterials, and an example showing liquid crystal-coated split-ring resonator metamaterials design. Reprinted from (Savo et al., 2014) with permission, Copyright@2014 Wiley-VCH. (E) A schematic showing phase change materials-based tunable THz metamaterials, and an example showing the phase change working mechanisms when an external optical pump was applied. Reprinted from (Pitchappa et al., 2019) with permission, Copyright@2019 Wiley-VCH. (F) A schematic showing 2D materials-based tunable THz metamaterials, and an example illustrating graphene-based tuning mechanisms by applying an external gate voltage to change the Fermi energy. Reprinted from with permission, Copyright@2018 Wiley-VCH.

Microelectromechanical system (MEMS) technology enables micro/nanoscale mechanical manipulation, which is suitable for meta-atom construction in the THz region, bringing various applications in THz functional devices. The reconfigurable MEMS metamaterials can be further classified by their actuator mechanisms, such as piezoelectric (Willatzen and Christensen, 2014; Amirkhan et al., 2020; Le et al., 2022), electrothermal (Lee and Wu, 2005; Lee and Yeh, 2005; Lee, 2005; Lee, 2006; Lee, 2007; Pitchappa et al., 2017), and electrostatic (Pitchappa et al., 2015a, 2015b, 2015c, 2016a, 2016c; Shih et al., 2017) (Pitchappa et al., 2021a, 2021b), and so on (Lee et al., 2005; Lee, 2005; Yeh et al., 2006). Combining with metamaterial resonator designs, the deformed structures can effectively modify the electromagnetic field distribution inside the resonators. Prakash et al. proposed a typical upward bending microcantilever as a metamaterial unit cell, which can tune the THz resonant spectrum when applied external bias, as shown in Figure 2A (Pitchappa et al., 2015c). The structure consists of top layer Al and dielectric layer Al_2_O_3_, which experienced deformation when electrostatic force was applied. A simplified model describing the tip-end displacement can be found in cantilever-related research (Chen et al., 1999). With the theoretically and experimentally demonstrated relationship between geometry and material parameters, the MEMS actuator combined with metamaterial resonator design can be fully utilized for THz modulators with a large tuning range and fast tuning speed.

### Thermal tuning mechanisms

The thermal tuning process directly leverages the temperature change of the environment, which induces structure or material property perturbation and in turn influences the resonance of the THz metamaterial resonators. In 2017, Prakash et al. proposed a reconfigurable thermal tuning microcantilever metamaterial device with an operating range from 77 K to 400 K, shown in Figure 2B (Pitchappa et al., 2017). The thermal expansion cantilevers experienced a beam deformation when the temperature changed, thereby shifting the resonance frequency, realizing frequency modulation in the THz range. Theoretical analysis of the out-of-plane deformation can be found in some research works (Zhou and Lee, 2017). Hence, with increasing temperature, the upward curvature is also increased and results in resonance change. Apart from thermal expansion of deformable structures, tuning the temperature-sensitive materials, such as vanadium oxide (Liu et al., 2012) and graphene (Cai et al., 2014), are also feasible for THz modulation applications. However, the temperature change is usually a slow process. Therefore, although with comparable modulation depth, the thermal tuning process can hardly be used for high-speed tuning applications.

### Micro/nanofluidic channels tuning mechanisms

Micro/nanofluidic is another interesting branch of tunable metamaterial devices for biosensing applications because most of the biomolecular processes happen in the aqueous environment (Li et al., 2017, 2018; Xu et al., 2020a, 2020b; Ren et al., 2021a, 2021b). The fluidic chamber integrated on metamaterial enables the transportation of solutions of different concentrations and components, and the contact with metamaterial hotspots. Owing to the high Q resonance of THz metamaterials, such nanofluidic chamber could be more sensitive to the concentration change of the targeted solution. Recently, Xu et al. proposed a dual-torus toroidal metasurface for THz microfluidic sensing application, as shown in Figure 2C (Xu et al., 2021a). The structure was fabricated by mechanically assembling the PTFE cap and bottom, containing the channel part with inlet and outlet solutions. Owing to the high Q factor induced by the toroidal mode in the THz region, when the concentration of inside fluidic changed, the effective refractive index also shifted, resulting in transmitted THz change with little perturbation in the concentration. Such a method could be used for labeled bio/chemical sensing applications (Shih et al., 2018, 2019).

### Liquid crystals tuning mechanisms

Liquid crystals, as birefringence material explored for the past few decades (Frank, 1958), are viable candidates for tunable metamaterials. When external AC bias was applied, the randomly aligned liquid crystal would become oriented with electric field lines, therefore, smoothly turning the refractive index as a function of an applied electric field, which in turn modifies the THz resonance (Shrekenhamer et al., 2013). In 2014, Savo et al. proposed a spatial light modulator (SLM) for THz applications utilizing liquid crystal metamaterial absorber, as shown in Figure 2D (Savo et al., 2014). The orientation of LC dimers was electronically controlled by biasing each metamaterial pixel with a fixed voltage, achieving binary control with a modulation depth of 70%. For continuous control, divergent frequency of AC signal could be applied to achieve smooth signal modulation with multiple states control (Ji et al., 2020). Leveraging such property, the smooth tuning mechanism enables the realization of multiplexed signal processing in THz communication, imaging, or THz waveplate application, especially applicable for active broadband devices.

### Phase change materials tuning mechanisms

Phase change materials will release or absorb sufficient energy when an external pump was applied, such as thermal, electrical, or optical source. For example, vanadium oxide will experience insulator-metal transition when the temperature reaches its critical temperature (Jerominek, 1993). Germanium-antimony-tellurium (GST) undergoes amorphous to crystalline phase change when temperature changes (Padilla et al., 2006). Leveraging the phase change phenomenon in THz photonics, the phase change process influences the conductivity change in the materials, which in turn modifies the THz resonance and controls the signal. Furthermore, this temperature-induced change could also be activated by an electrical or optical pump, therefore, enabling the realization of ultrafast applications. In 2019, Pitchappa et al. proposed GST-integrated THz metamaterial devices for active tuning applications, as shown in Figure 2E (Pitchappa et al., 2019). The conductivity change in the GST layer comparably influenced the confined electrical field in the metamaterials structures and manipulated both Fano resonance and dipole mode resonance. Besides, the modulation speed could reach the GHz level under the optical stimulus. This could be further improved by exploring materials with faster response in recent studies (Tan et al., 2021), promising future ultra-high-speed devices for THz on-chip communication.

### Two-dimensional materials tuning mechanisms

Two-dimensional (2D) materials refer to crystalline solids consisting of a single layer of atoms, which are suitable for THz applications due to their high carrier mobility, enabling a fast response rate for THz applications (Tassin et al., 2013; Sizov, 2018). Therefore, leveraging 2D materials for THz modulation could benefit from its low lifetime and high mobility of carriers. Among all the 2D materials, graphene has been reported many times for THz applications due to its high response rate and low noise, especially for THz detectors (Vicarelli et al., 2012; Mittendorff et al., 2013; Stantchev et al., 2020). In addition to that, the mechanisms of tunable graphene THz devices are also varied, such as bolometric (El Fatimy et al., 2016; Muraviev et al., 2013; Lara-Avila et al., 2019), plasmonics (Tong et al., 2015; Bandurin et al., 2018; Salamin et al., 2019), and photo-thermoelectric (Cai et al., 2014; Viti et al., 2020). In 2018, Kindness et al. proposed an active THz metamaterial array with graphene for continuous resonance frequency tuning, as shown in Figure 2F (Kindness et al., 2018). By changing the graphene conductivity when external bias was applied, the resonance frequency, as well as the coupling between resonances were tuned, realizing amplitude and frequency modulation in the THz region. Recently, there have also been many explorations of other 2D materials for THz modulator applications with faster modulation speed and higher modulation depth (Li et al., 2020b; Chen et al., 2021). However, one major limitation is the large-scale fabrication process of 2D materials, which promises the commercial and development of the 2D materials for THz commercial applications.

## THz reconfigurable metamaterials with single resonance

### Localized surface plasmon resonance, Mie resonance, and temporal coupled-mode theory

Before we discuss different tuning dimensions of THz metamaterial modulators, we briefly introduce the principles of localized surface plasmon resonance (LSPR), Mie resonance, and temporal coupled-mode theory (TCMT) that will be required to follow this review. These three theories describe the THz electromagnetic resonance from the aspect of near-field and far-field properties. A more in-depth introduction can be found in several existing reviews (Maier and Atwater, 2005). This section starts from the physical principles of plasmonic resonances and the relationships between nanostructures, followed by several examples regarding different tuning dimensions and their performance, respectively.

THz modulators behave like a resonance system, where many theories can be used for explaining the working mechanisms, such as the lumped equivalent circuit model (Engheta et al., 2005), temporal coupled-mode theory (Bertolotti, 1985), interference theory (Saleh and Teich, 2007; Chang et al., 2020a, 2020b), and so on. In this review, we will introduce the localized surface plasmon resonance (LSPR), Mie resonance, and temporal coupled-mode theory (TCMT). The former two resonant systems could give an explicit explanation of the near-field effect of plasmonic and dielectric structures, respectively. While the last is widely used for illustrating how the near-field properties affect the far-field behaviors.

We start from the LSPR phenomenon. When an external electromagnetic field is applied to a plasma region, the phenomenon of collective oscillation of electrons and photons is quantized as plasmons (Maier et al., 2001). Plasmons can be divided into bulk plasmon and surface plasmons concerning the orientation of oscillation, where the longitude-generated wave is called bulk plasmon, and the transverse wave is known as surface plasmon. Therefore, owing to the continuous condition of tangential electric fields, external electromagnetic waves could only activate the surface plasmon. Owing to the free electron of the noble metal behaving like a gas of charge carriers, surface plasmon can be excited on the surface of such materials. Furthermore, we could leverage Drude’s model to describe the dielectric properties of noble metals (Bade, 1957). By solving the equation of motion of plasma sea subjected to an external electric field, the dielectric function of free electron gas located on the surface of noble metals can be expressed below:

(Equation 1) $$\varepsilon \left(w\right)=1-\frac{\omega_{p}^{2}}{\omega^{2}+i\gamma \omega }$$

where *ε(ω)* denotes the dielectric function, *ω* denotes the frequency of incident electromagnetic waves, *γ* denotes the characteristic collision frequency, and *ω*_*p*_ denotes the plasma frequency, which is related to the electron density and differs from materials. When the incident frequency nearly equals the plasma frequency, the real part of the dielectric function *Re{ε(ω)}* is near equal to 0. Therefore, if we apply the condition that the tangential electric field must be continuous near the metallic surface, we will find that the intensity of the near-field becomes ultimately high. This enhancement of the near-field intensity is known as localized surface plasmon resonance (LSPR), and frequency is called the resonance frequency. Owing to the enhancement of the electric field, the light behaves as if it were confined into the subwavelength region where the plasmons are generated. It is this confinement of light that forms a resonance cavity. However, it is not difficult to find that the resonance frequency of such cavity is near the plasma frequency, while the plasma frequency is determined by the electron density. Therefore, an efficient way of tuning the resonance frequency is to artificially change the electron density, which is, to design the plasmonic structures. Such artificially designed structures are also known as plasmonic metamaterials. For example, modeling the design principles of half-wave dipole antennas in traditional radio and microwave frequencies, the designed subwavelength nanorod structure (also called nanoantenna) follows a similar rule when illuminated by linear polarized light, given by (Neubrech et al., 2017):

(Equation 2) $$\lambda =\frac{2L}{m}na_{1}+a_{2}$$

where *L*, *m*, and *n* are the physical length of the nanoantenna, model number, and refractive index of a surrounding medium, respectively. The constant *a*_*1*_ and *a*_*2*_ represent the relationship between the structure and material, and the phase associated with the reflection at the structure end, respectively. Therefore, a more intuitive conclusion is that the length of the nanorod-like structure and the environment refractive index could affect the resonance frequency at a confined resonance cavity. This conclusion is also similar to the conventional six-wall resonance cavity, where the shape of the box, as well as the stuffed material, all contribute to the resonance shift. Apart from the nanorod structure, another typical plasmonic structures that are widely used as metamaterial unit cells are the split-ring resonator (SRR) (Zhou et al., 2005). Compared with the nanorod, SRR were first put forward as magnetic resonators, where the metallic loop structure could behave like a magnetic dipole. Applying the lumped circuit model, the folded metallic wire and the induced gap function as an inductor and a capacitor, respectively. Therefore, modeling the parallel resonant L-C circuit, the resonant frequency and strength are dependent on the full length of SRR as well as the gap distance (Engheta et al., 2005). Furthermore, owing to the strong confinement of the electric field power within the gap region, asymmetric SRR structures could enable a trapped mode of the structure, where high Q-factor resonance can be induced for tunable metamaterial designs (Fedotov et al., 2007). Leveraging such design principles, the plasmonic nanoantennas are widely used for chemical sensing (Shih et al., 2019; Wei et al., 2019; Zhou et al., 2020; Chang et al., 2021; Ma et al., 2021; Zhou et al., 2021c; Liu et al., 2021), radiation detection (Salamin et al., 2019; Wei et al., 2020, 2021), imaging (Huang et al., 2013; Zheng et al., 2015), and silicon photonics applications (Wei and Lee, 2019; Chen et al., 2018; Ren et al., 2021a, 2021b).

Dielectric materials are also applicable for resonators (Petosa and Ittipiboon, 2010) (Higuchi and Tamura, 2003) different from the metallic structures, where the permittivity ε_r_ is smaller than zero, which also brings the dissipation. When the electromagnetic wave illuminated onto a dielectric structure, part of the wave will be transmitted through the structure due to the positive dielectric constant, promising higher transmission efficiency and lower dissipation than plasmonic structures. Therefore, dielectric resonators utilized materials that have higher index, such as silicon, germanium, or tellurium (Jahani and Jacob, 2016). Furthermore, the resonant system can also be described by Mie theory (Wriedt, 2012). When the frequency of the electromagnetic waves is below or near the bandgap frequency of the patterned dielectric resonators, a magnetic dipole and an electric dipole will be excited simultaneously. The magnetic and electric dipoles are also called first Mie resonance and second Mie resonance, respectively (Dobson, 1984). Compared with the plasmonic structures, the first Mie resonance presents circular displacement currents, which assembles the SRR structure, while the second Mie resonance behaves like nanorod structures. However, the SRR structure could only work at low frequencies, while the dielectric magnetic dipole is also effective in optical frequencies. Therefore, for single resonant elements, although the dielectric resonators could not support the near-field enhancement as the plasmonic ones, the Mie resonance also enables the excitation of electric and the magnetic dipole at optical frequencies with lower dissipation. Furthermore, if the all-dielectric resonators are coupled, they can also enable similar near-field effect as the plasmonic structures, such as hot-spots for near-field scanning optical microscopy or chemical sensing (Bakker et al., 2015) (Tittl et al., 2018). However, compared with plasmonic resonators, even the coupling could generate high Q-factor resonance, or the magnetic field coupling, the far-coupling is usually weaker. In addition to that, the fabrication process of spherical-shaped Mie resonators is far beyond the conventional lithography and reactive ion etching (RIE) techniques. Therefore, cylinder-shaped or rectangular-shaped dielectric resonators should be given first priority for on-chip devices.

Up to now, we have given a brief introduction to LSPR and Mie resonance. Furthermore, engineering the structures into the metamaterials could enable the tuning of the resonance frequency. However, how to quantitatively ensure that the near-field effect influences the results of the far-field spectrum, where transmission, reflection, and absorption are always used for describing a resonance system, is still ambiguous. Therefore, we would like to introduce temporal coupled-mode theory (TCMT), which provides insights into the resonance system and reveals significant parameter optimization rules for metamaterial resonators. Besides, TCMT could also be applied for dual resonance systems, such as electromagnetically induced transparency (EIT)-like or Fano-like resonance (Luk’yanchuk et al., 2010). In this section, we will introduce the TCMT model for a single resonance system first, while the application for a dual resonance system will also be covered in the next section. The expression of TCMT for a single resonance system could be given by the following general equations (Bertolotti, 1985):

(Equation 3) $$\frac{d}{dt}P=j\omega_{0}P-\left(\gamma_{r}+\gamma_{a}\right)P+\sqrt{\gamma_{r}}s_{in}$$

(Equation 4) $$s_{t}=s_{in}-\sqrt{\gamma_{r}}Ps_{r}=\sqrt{\gamma_{r}}P$$

(Equation 5) $$T={\left|\frac{s_{t}}{s_{in}}\right|}^{2},\;R={\left|\frac{s_{r}}{s_{in}}\right|}^{2}$$

where *P* is the amplitude of plasmonic mode, *w*_*0*_ represents the resonance frequency of plasmonic mode, while *γ*_*r*_ and *γ*_*a*_ denote the radiative and absorptive losses of plasmonic structures. *T* and *R* denote transmission and reflection spectra, which are related to the amplitude of incident light (*s*_*in*_), reflected light (*s*_*r*_), and transmitted light (*s*_*t*_). By substituting the Equations 3 and 4 into Equation 5 and replacing *d/dt* term with time-harmonic term *jw*, the far-field spectra of a given frequency *w* can be determined by

(Equation 6) $$T\left(\omega \right)={\left|\frac{j\left(\omega -\omega_{0}\right)+\gamma_{r}}{j\left(\omega -\omega_{0}\right)+\left(\gamma_{a}+\gamma_{r}\right)}\right|}^{2}$$

(Equation 7) $$R\left(w\right)={\left|\frac{j\left(\omega -\omega_{0}\right)+\left(\gamma_{a}-\gamma_{r}\right)}{j\left(\omega -\omega_{0}\right)+\left(\gamma_{a}+\gamma_{r}\right)}\right|}^{2}$$

From Equations 6 and 7, it can be noticed that the two key parameters influencing the far-field spectra are frequency and loss. Besides, when the incident frequency *w* matches the resonance frequency of plasmonic mode *w*_*0*_, the amplitude of the transmission (or reflection) will reach its maximum (or minimum) value, which is also the condition of LSPR. Therefore, the TCMT theory could bridge the relationship between near-field coupling and far-field spectra. Furthermore, manipulating the relationship between *γ*_*r*_ and *γ*_*a*_ is also effective in tuning the far-field spectrum, especially in their influences on the amplitude. In several recent research papers, it has been demonstrated that the ratio between *γ*_*r*_ and *γ*_*a*_ plays a significant role in sensing applications (Wei et al., 2019; Xu et al., 2020a; Li et al., 2021). A more intuitive comment on these two methods is to directly compare them with the FM and AM modulation in radio frequencies, where the tuning of resonant frequency models FM, while the tuning of losses assembles AM. Therefore, the modulation through *w* could get rid of interferences over the working frequency band, while the modulation through *γ*_*r*_ and *γ*_*a*_ could work on wider frequencies.

In the next few parts of this section, we will separate the tuning mechanism into frequency tuning and loss tuning to show how the parameters influence the far-field performance. Based on that, we will further introduce a method of decoupling these two coefficients and make them work independently.

### Frequency-tunable THz metamaterials

**Figure 3:**
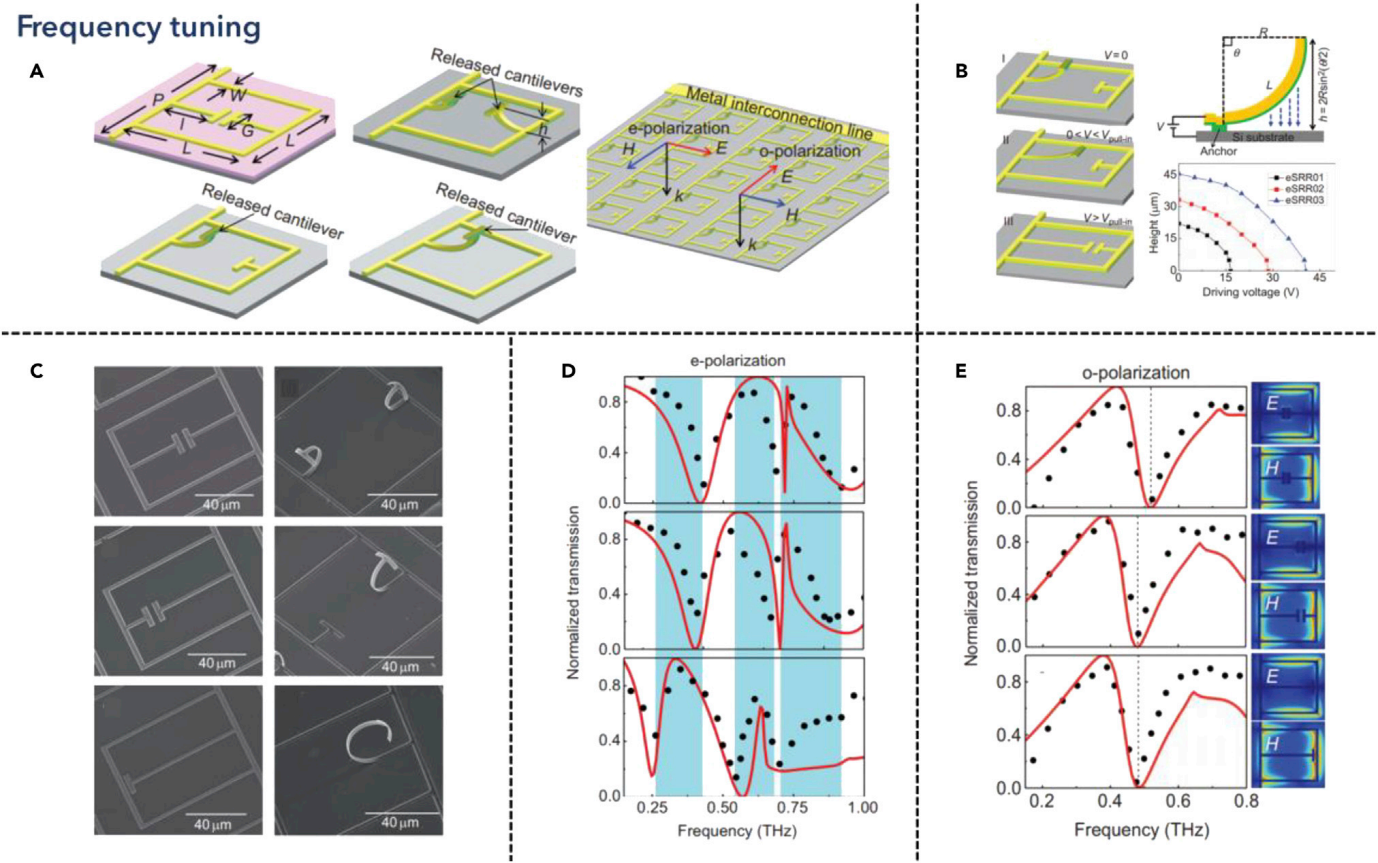
Frequency tunable THz metamaterials Reprinted from ref (Ma et al., 2014) with permission. Copyright@2014 Springer Nature. (A) Different unit cell and arrayed metamaterials design with various incident polarizations. (B) Theoretical analysis of the relationship between applied voltage and released height. (C) Scanning electron microscope (SEM) image of the released and non-released unit cells. (D) Normalized transmission under e-polarized incidence, with varied released height. (E) Normalized transmission and near-field intensity of different unit cells.

Frequency tuning is one of the most intuitive methods of modulating THz waves. As we mentioned in the last part, the artificially constructed metamaterials can tune the resonance frequency, which leverages the similar principle of dipole-antenna design. Therefore, one of the most efficient methods of frequency tuning is to change the effective electrical length of the environment. Owing to almost all the nanostructures being patterned lithographically, the geometry of the plasmonic structure is difficult to modify once fabricated. Therefore, one efficient way is to leverage MEMS technology to dynamically tune the structures. Ma et al. proposed such multi-band tunable metamaterials using a reconfigurable electric SRR array, as shown in Figure 3 (Ma et al., 2014). When illuminated with THz light, the proposed SRR structures were metamaterial resonators, which could be equivalent to a lumped circuit model in THz, where the confined electric field was localized in the in-plane gap, shown in Figure 3A. Therefore, a further modification was to combine the cantilever structure with the SRR, which will be released to a certain height after actuation, in turn changing the resonance condition of SRRs, and completing the active tuning process, depicted in Figure 3B. It is worth noting that the actuation process was done by applying an external DC bias, where for different structures, the maximum released height differs at the same actuation voltage. Besides, considering the tuning range of a single SRR structure, the height cannot be released from infinity to zero after the fabrication and actuation process, limiting its working frequencies. Hence, the unreleased SRRs were initially varied from others, where three different types were demonstrated and released independently, as shown in Figure 3C. Furthermore, such a proposed structure also presented a double resonance state when illuminated with different polarizations, adding one more tunable dimension to the device. When resonating at around 0.45 THz, the tunable range could reach the highest value of 0.25 THz, as shown in Figures 3D and 3E. Consequently, the structure fully leveraged the wide tunable range provided by MEMS technology, where the combination of electrostatic force-actuated cantilevers and metamaterial resonator acts as a promising platform for the THz-tunable modulators. In addition to that, several design details are also revealed to have potential in improving performance. On the one hand, from the mechanical part, the cantilever shape could be further optimized to achieve larger deformation, for which it would be easier to actuate and reach a larger tuning range. Besides, the material of the cantilever should also be properly chosen, where Young’s modulus and the thermal expansion coefficient both contribute to the curvature of the beam. On the other hand, from the optical part, the resonance should have a higher Q factor, which compromises higher confinement of electric field power, and therefore higher selectivity toward frequency.

For the mechanical part, Ho et al. proposed an omega-ring cantilever-structured THz metamaterial which could be actuated electrothermally (Ho et al., 2014). This omega-ring design was composed of the same materials, however, simply changing the moving part from the nanorod-shaped to the ring-shaped structure provided better mechanical characteristics, therefore, only actuated by an external DC bias of 5 V and a driving current of 100 mA, the metamaterial THz modulator could also achieve a tuning range of 0.30 THz. Following similar designing rules, Pitchappa et al. proposed subwavelength MEMS cantilevers with optimized nanorod structure, reaching a switching range around 0.29 THz and a modulation depth around 60% at 0.59 THz (Pitchappa et al., 2015c). In the same year, Pitchappa et al. also proposed another tunable multi-band metamaterial design with preserved isotropy, where an octagon-shaped unit cell geometry was put forward to preserve the symmetry, thus keeping the polarization response independent (Pitchappa et al., 2015b). The switching range was only 0.16 THz, but the modulation depth was significantly improved to 80% at 0.57 THz. Apart from improving the tuning performance of THz metamaterial resonators, another research topic is to search for the functionalities, especially from the aspect of polarization. Kan et al. proposed a chiral metamaterial for THz polarization modulation using MEMS spiral structures, enabling the switching of circularly polarized light through an enantiomeric structure (Kan et al., 2015). The changing of deformation direction provided an intuitive method of constructing three-dimensional chiral structures, which was important for enhancing the optical chirality due to its spatial distribution of electric field (Mun et al., 2020). Therefore, unlike traditional 3D chiral metamaterials, only with in-plane lithography patterning, it is still feasible to fabricate 3D chiral structures for circularly polarized light manipulating. Another work conducted by Cong et al. utilized the same working mechanism of electrically tuning the structure; however, the method of constructing the chirality was different, where the deformation of the cantilever beam changed the incident angle of THz light, and in turn influenced the chirality (Cong et al., 2019). Therefore, compared to the previous work, this optically induced chirality was extrinsic, while the structure itself was achiral. After the deformation of the beam, the illuminated THz light onto the structure was not normal, and an asymmetric-induced optical chiral term was generated.

For the optical part, one way to improve the quality factor is to leverage different modes. In 2007, Fedotov et al. proposed sharp trapped-mode resonance in planar THz metamaterial with broken structural symmetry (Fedotov et al., 2007). The Q factor was one order of magnitude larger than the typical value of dipole mode. Therefore, planar structures with symmetry-broken structures opened a new way for narrow resonance linewidth, which had potential in frequency tuning due to its higher selectivity. However, to preserve the designed asymmetry structures, traditional MEMS technology leveraging electrostatic force for active tuning may not be the best choice, where the preserved planar geometrical asymmetry cannot be precisely modified, which may influence the quality factor. Therefore, more advanced solutions are to tune the electromagnetic properties of the surrounding materials. Liu et al. proposed an insulator-to-metal transition in vanadium oxide when the environment temperature exceeds its critical temperature, which was induced by the THz pulse (Liu et al., 2012). Although this work did not reveal the possibility of modulating THz response inversely, such phase change materials were demonstrated to have such potential applications. In 2019, Pitchappa et al. proposed a similar active frequency tuning method using GST (Pitchappa et al., 2019). Compared with vanadium oxide, GST changes its phase from amorphous to crystalline when an external pulse is applied, but with a faster response rate. Therefore, GST is more suitable for modulation applications. Besides, apart from phase change materials, tuning the conductivity of two-dimensional materials also provides insights into the active tuning of frequency in the THz region. Recently, Xu et al. also proposed stretchable THz metamaterials, where the stretching of flexible substrate modified the confinement of electric field in the high Q-factor parabolic-shaped metamaterial resonator, thereby tuning the frequencies (Xu and Lin, 2021). Although the tuning range of such stretchable deformation was smaller than previous electrostatic actuated cantilever-based metamaterials, the flexibility of the device was promising for wearable applications (Xu and Lin, 2019).

In summary, as an effective way of modulating THz light, frequency tuning indicates the modifying of parameter *w* in the TCMT model, provides necessary development and potential solutions of frequency modulation in the THz range. Besides, the modulation performance could be both improved mechanically and optically, where the former could enhance the tuning range and the latter could minimize the tuning steps. In addition to that, from the aspect of fabrication, the MEMS technology outperforms the material tuning methods due to its CMOS compatibility. Therefore, regarding future development, researchers are encouraged to explore faster tuning speed and wider tuning range, while considering the availability and feasibility in the fabrication process.

### Amplitude-tunable THz metamaterials

**Figure 4:**
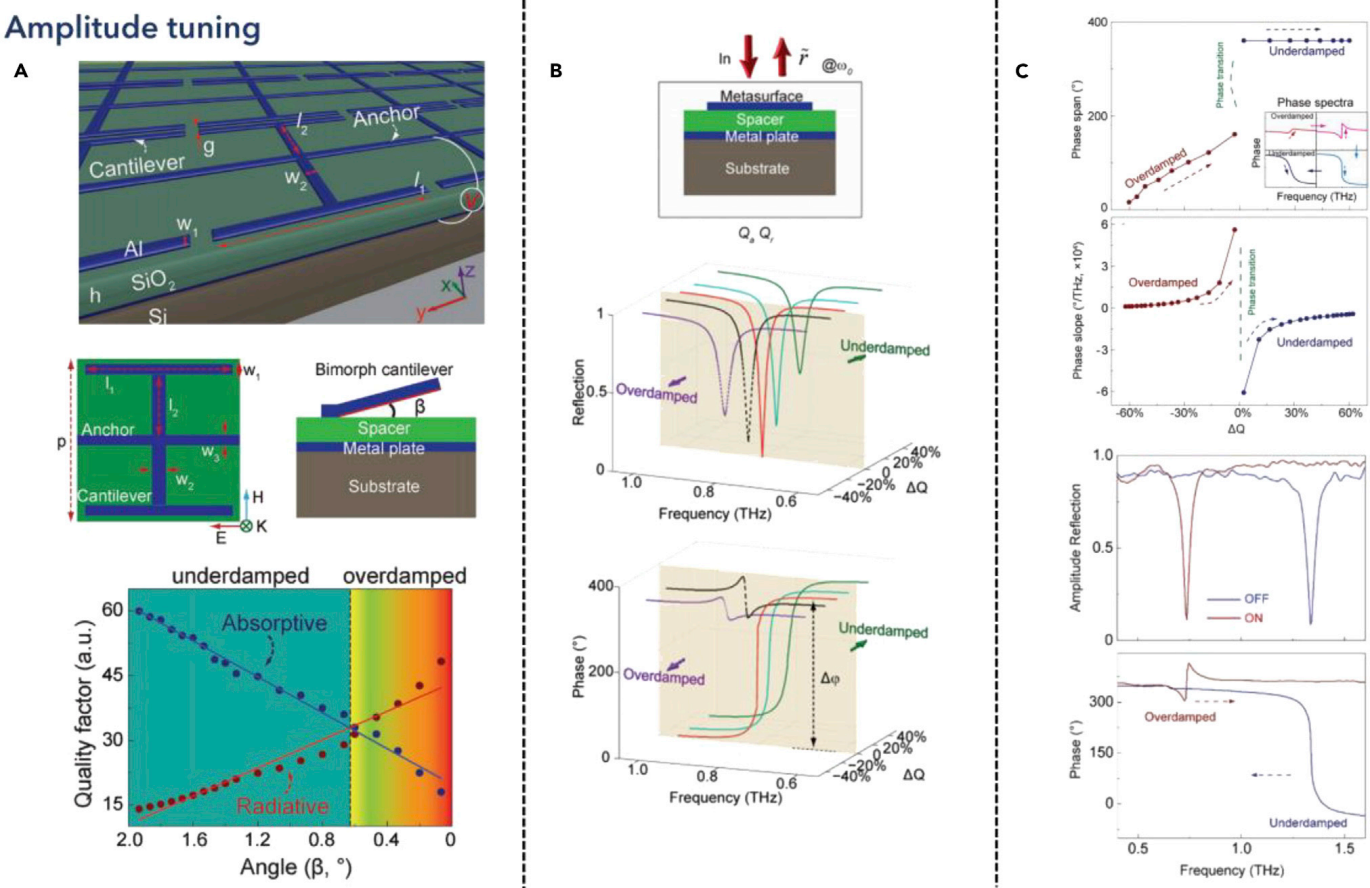
Amplitude tunable THz metamaterials Reprinted from ref (Cong et al., 2017b) with permission. Copyright@2017 Wiley-VCH. (A) Cantilever-based metal-insulator-metal (MIM) design for tailoring the radiative and absorptive quality factors. (B) Model of MIM unit cell described by coupled-mode-theory. (C) Phase change properties in the underdamped and overdamped regimes, and the measured amplitude tuning results.

Amplitude modulation plays another important part in THz active tuning. Unlike the mechanisms of frequency tuning, where the resonance simply shifts to shorter or longer wavelengths, amplitude tuning emphasizes how to tune the coupling strength of the resonance. Similar to the AM modulation in radio frequency, amplitude tuning suffers the problem of interference among frequencies. However, the flexibility tuning of the amplitude enables the quantification of multiple states within one frequency, providing one significant dimension to adjust the resonance for analog applications. In 2017, Cong et al. proposed a THz metamaterial leveraging the active phase transition process via loss engineering, as shown in Figure 4 (Cong et al., 2017b). The structure utilized MEMS technology, where a conventional bimorph cantilever was used for electrostatic actuation. When external DC bias was applied to the device, an increased height induced angle between the cantilever and the spacer *β* could adjust the quality factor of the resonator, where the absorptive and the radiative dissipation could be dynamically tuned and result in different quality factors. Besides, as the deformation angle changed, the absorptive and the radiative Q factors experienced opposite trends, while these two coefficients reached the same quality factor at a certain bending angle around 0.6°, as depicted in Figure 4A. To further comprehend the physical meaning of the radiative and absorptive quality factors, a far-field spectrum illustrated that the metal-insulator-metal structure achieved its highest Q factor at the point where the absorptive factor equaled the radiative factor at around 0.8 THz. Therefore, the change of the reflection depth reached its maximum value. For the difference between the underdamped and overdamped region, the reflection phase spectra illustrated that the total difference of the phase of the underdamped region is much larger than the overdamped region, shown in Figure 4B. An explanation of this phenomenon was that a phase transition process occurred in this MIM region, shown in Figure 4C. Further analysis showed that it was the released height of the cantilever beam that determined the quality factors, which essentially decided the effective spacer thickness, and influenced the coupling strength between the two metal layers. Consequently, the amplitude change of reflection or transmission spectra was significantly correlated with the loss of radiative loss and absorptive loss of the metamaterial resonator system, respectively. Tuning the resonant system to a critical point where the dissipation of radiative and absorptive matches could promise higher modulation depth and anomalous phase transition. Similar to frequency modulation, there are two aspects that can be further explored. One is to investigate the phase transition phenomenon and its related applications in the THz region. Zhao et al. proposed an electromechanically tunable THz metasurface for transmissive waveplate application (Zhao et al., 2018). Utilizing the phase transition of different polarizations of the incident light, the device presented various transmission spectra, which could be further modified by applying different external DC biases. Liu et al. proposed subwavelength metasurface resonators for wavefront manipulation using a refined structure (Liu et al., 2019a). A bonding process between two samples was implemented to induce a nanogap vertically, replacing the metal-insulator-metal layered devices. Therefore, the phase transition could be induced through the coupling between the samples, which in turn determined the steering angle of reflected light. Another approach is to engineer the loss of metamaterials. To overcome the ohmic loss of plasmonic structures, Ji et al. proposed a grating structured dielectric metasurface, tuned by liquid crystal spacer between the silicon substrate and silica top surface (Ji et al., 2020). Tuning the frequency of external AC bias, the birefringence of liquid crystal could be tuned and thus presented different refractive index and absorption coefficient, which modulated the magnitude ratio in THz spectra. Cong et al. also proposed spatiotemporal dielectric metasurfaces based on Kerker’s condition, which exhibited unidirectional propagation of light by tuning the quality factor of the electric and magnetic dipole to the critical damping point under different power (Cong and Singh, 2020). The low-loss feature of dielectric metamaterials provided ultra-high Q factor and tunability with perfect degeneracy of electric dipole and magnetic dipole. From another aspect, to change the field confinement between the resonator and surrounding materials, one typical method is to change the conductivity of materials, which in turn influences the free carriers in the material, and thereby modifies the near-field confinement intensity, as well as the relationship between radiative and absorptive losses. Ju et al. proposed tunable THz metamaterials controlled by the graphene-plasmonics structure, where the Fermi energy was tunned by applying external DC bias, changing the carrier concentration, and in turn affecting the free carrier absorption (Kindness et al., 2018). Recently, Than et al. also proposed an active control process of THz Quasi-BIC with ultra-high Q factor leveraging germanium with a short switching time of 7 ps (Tan et al., 2021). This work reveals the perspective of future development of THz amplitude tuning using dielectric metamaterials, where leveraging the Quasi-BIC design of metamaterials could reach higher quality factors while exploring materials with a faster response rate could become more promising in future THz frequency modulators.

In summary of this part, the engineering of radiative and absorptive loss played an important part in controlling the coupling strength of a THz resonator system, where at the intersection of the underdamped and overdamped regions, the system achieves the largest preservation of power. Besides, a phase transition could also be observed, which is potential in beam steering and wavefront manipulation. Moreover, to enhance the Q factor, replacing the plasmonic structure with dielectric ones could further restrict the loss and enable higher amplitude modulation performances.

### Hybrid magnitude-frequency tunable THz metamaterials

**Figure 5:**
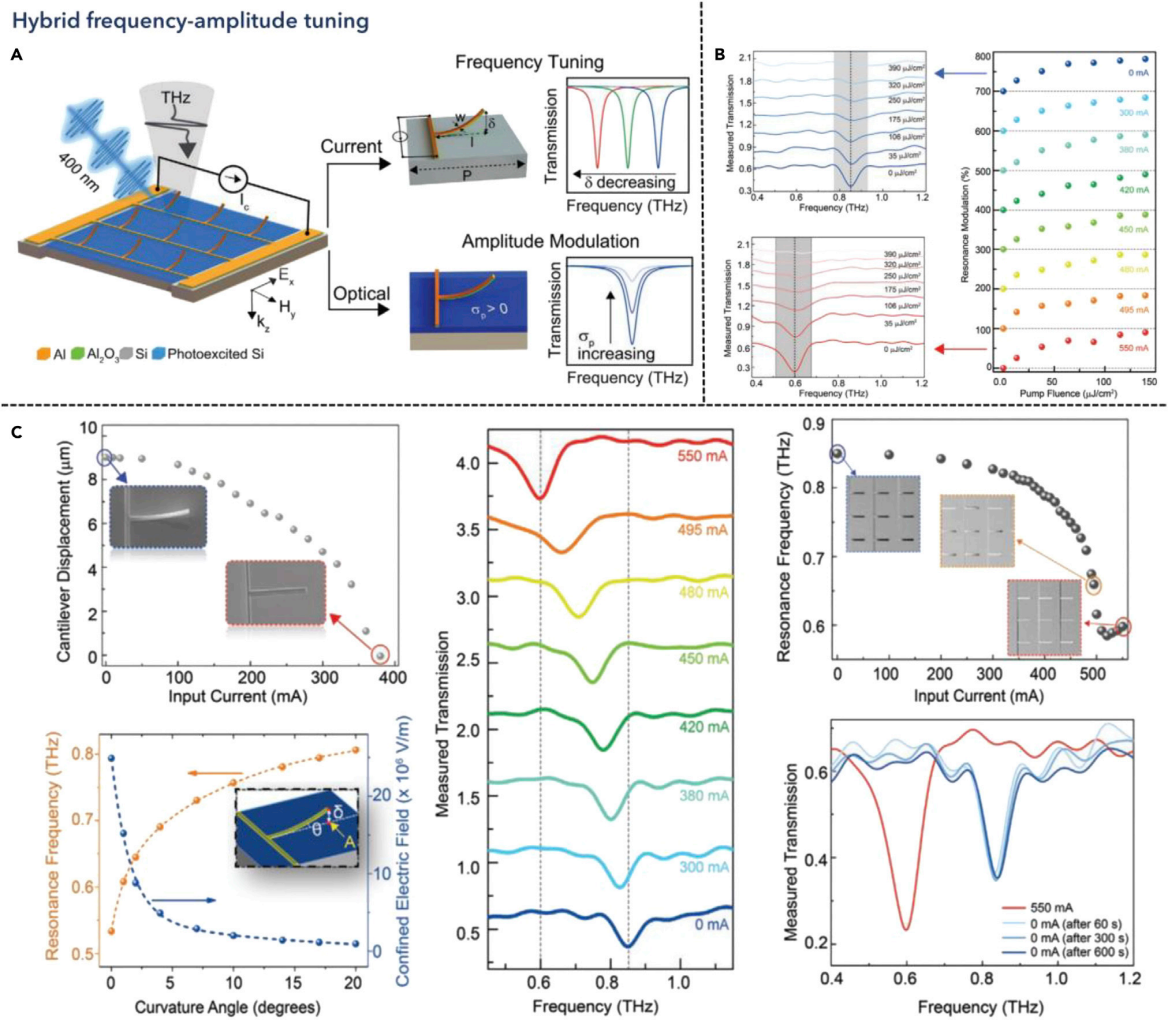
Hybrid amplitude-frequency tunable metamaterials Reprinted from ref (Pitchappa et al., 2020) with permission. Copyright@2020 Wiley-VCH. (A) Schematic representation of the metamaterial with optical and electrical tuning processes regarding amplitude and frequency modulation. (B) Measured THz transmission spectra of the metadevice with different pump fluence. (C) Measured THz transmission spectra of the metadevice with varying tip displacement.

A more advanced approach to manipulate the THz wave is to combine the magnitude and frequency tuning, forming a hybrid modulation platform. Multi-domain reconfiguration has been explored with multiple functions and gaining significant research attention (Liu et al., 2016; Shabanpour et al., 2020; Tian et al., 2020; Cui et al., 2020). Engineeringly, this provides one more dimension of active tuning at the same platform. Scientifically, from the perspective of the TCMT model, independently tuning could be a solution to decouple loss and frequency for advanced applications. Recently, Pitchappa et al. proposed a microcantilever metamaterial structure that realized the frequency and amplitude modulation, where different mechanisms were activated optically and electrically (Pitchappa et al., 2020). Furthermore, the ultrafast modulation with the narrowband operation of metamaterials, as a long-standing issue, was also demonstrated by integrating MEMS cantilever with an ion-irradiated silicon substrate. The electrostatic-actuated microcantilever shifted the resonance frequency when deformed, while the photoexcited silicon layer changed the intensity of transmitted light under the optical stimulus, due to its photoconductive effect, as shown in Figure 5A. Increasing the pump fluence, the change in the conductivity of silicon would generate free carriers, cloaking the confined near-field resonance. Thus, the transmissive resonant dip would broaden and gradually vanished, marking a maximum modulation depth near 100%. Besides, to explore the ultrafast response performance, a time delay between optical pump and THz probe was further studied, where lower time delay was required to achieve better modulation depth, and a recovery time was observed at around 400 ps, as shown in Figure 5B. Apart from the amplitude modulation, frequency tuning was implemented electrically by applying another DC bias to the cantilever beam, where the electrostatic force induced different released heights, changing the resonance frequency with a tunability of 0.25 THz when applying an input current of 550 mA, as depicted in Figure 5C. Moreover, the cantilever structure recovered to almost 90% of the initial state of beam displacement after stopping the current input. Therefore, combining MEMS technology and photoconductive materials could potentially realize high-speed THz communication for multi-dimensional controllable devices. Owing to the ultrafast response, exploring different tuning dimensions with photoactive materials has become another research topic. Hu et al. proposed an ultrafast all-optical switching utilizing spatiotemporal THz metasurface (Hu et al., 2021c). The electrically controlled vanadium oxide provided a frequency shift due to the phase change process, while the photoconductivity was controlled when the pump fluence changed, modulating the amplitude of the transmission dips. Furthermore, the recovery time was also decreased to around 16 ps. However, owing to the slow phase change process of vanadium oxide, the frequency tuning process could be further improved. Therefore, following this work, the authors also came up with other two recent studies, where polarization and incident angle-tuned mechanisms were explored, respectively, to realize faster frequency modulation response (Hu et al., 2021a, 2021b). However, the photoconductive recovery time increased up to 700 and 2000 ps, respectively. Therefore, a trade-off needs to be further discussed for future applications.

## THz reconfigurable metamaterials with multiple resonances

### Temporal coupled-mode theory for dual resonant system

Different from the single resonant system, a dual-mode resonant system provides one more dimension for modulators. Combining various modulating mechanisms into two resonance frequencies could enable the realization of multi-functional devices. However, the design principles are usually more complicated compared with single resonant systems. Therefore, a tradeoff between different systems needs to be further discussed from the perspective of applications.

Owing to the existence of another resonance mode, the TCMT model should also include another coefficient that describes the relationship between two modes. Therefore, a more explicit expression of the TCMT model is (Wei et al., 2019)

(Equation 8) $$\frac{d}{dt}P=j\omega_{0}P-\left(\gamma_{r}+\gamma_{a}\right)P+j\mu M+\sqrt{\gamma_{r}}s_{in}$$

(Equation 9) $$\frac{d}{dt}M=j\omega_{m}-\gamma_{m}M+j\mu P$$

(Equation 10) $$s_{t}=s_{in}-\sqrt{\gamma_{r}}Ps_{r}=\sqrt{\gamma_{r}}P$$

(Equation 11) $$T={\left|\frac{s_{t}}{s_{in}}\right|}^{2},\;R={\left|\frac{s_{r}}{s_{in}}\right|}^{2}$$

where an extra resonating mode *M* was introduced to this expression. *w*_*m*_ and γ_m_, represents the resonance frequency and the absorptive loss of the mode *M*, respectively. Besides, the coefficient *μ* describes the coupling strength between mode *P* and mode *M*. Comparing with the single resonant model shown from Equations 3 to 5, the dual resonant model includes another mode and its related absorptive loss, with an extrinsic coefficient showing the relationship between two modes. A more intuitive expression for the transmission and reflection coefficient is

(Equation 12) $$T\left(\omega \right)={\left|\frac{j\left(\omega -\omega_{0}\right)+\gamma_{a}+\frac{\mu^{2}}{j\left(\omega -\omega_{m}\right)+\gamma_{m}}}{j\left(\omega -\omega_{0}\right)+\left(\gamma_{r}+\gamma_{a}\right)+\frac{\mu^{2}}{j\left(\omega -\omega_{m}\right)+\gamma_{m}}}\right|}^{2}$$

(Equation 13) $$R\left(\omega \right)={\left|\frac{\gamma_{r}}{j\left(\omega -\omega_{0}\right)+\left(\gamma_{r}+\gamma_{a}\right)+\frac{\mu^{2}}{j\left(\omega -\omega_{m}\right)+\gamma_{m}}}\right|}^{2}$$

It can be noticed that another mode-related term includes the resonant frequency *w*_*m*_, the absorptive loss γ_m_, and the coupling strength *μ* all appear in the spectrum coefficient. Therefore, a more complicated and comprehensive TCMT model is there to describe any dual-mode resonant system such as Fano-like resonance and EIT-like resonance. Besides, as mentioned in the last section, the incident frequency should match the resonant frequency of the metamaterial resonator to reach better performance for a single resonator system. Similarly, when the resonant frequency of the dual-mode system matches the incident frequency, which is *w* = *w*_*m*_ *= w*_*0*_, the highest performance is also achieved. Furthermore, if we also assume that the coupling between these two modes is weak, meaning that the modes can be tuned independently, the expressions of the different reflection and transmission signal can be obtained:

(Equation 14) $$\Delta T=\frac{2\gamma_{a}\gamma_{r}}{{\left(\gamma_{a}+\gamma_{r}\right)}^{3}}\frac{\mu^{2}}{\gamma_{m}}$$

(Equation 15) $$\Delta R=\frac{-2{\gamma_{r}}^{2}}{{\left(\gamma_{a}+\gamma_{r}\right)}^{3}}\frac{\mu^{2}}{\gamma_{m}}$$

The expression of *△T* and *△R* is different, where the proportion of *△T/△R* is equal to *γ*_*a*_*/γ*_*r*_. The absorptive loss mainly comes from the ohmic loss of plasmonic structures; therefore, it is relatively robust than the radiative loss. As a result, for the bright mode, the value of *γ*_*r*_ is usually higher than *γ*_*a*_. Consequently, *△R* will also be larger than *△T*. In the contrast, *△T* will become larger than *△R* for the dark mode resonance. Therefore, how to optimize and tune the relation between different resonant modes will play an important role in dual resonant systems, which is the key factor of achieving better modulation depth and tuning rate for THz modulators.

Overall, to generate the dual-mode resonance, one way is to design the two resonance frequencies and the losses independently with an individual method of tuning, which could be applied for multi-functional devices with multiplexed applications in frequency, while the other method is to deliberately overlap the resonances, where the coupling strength provides another dimension for controlling the resonance condition.

### Dual resonance modulators in weak coupling regions

**Figure 6:**
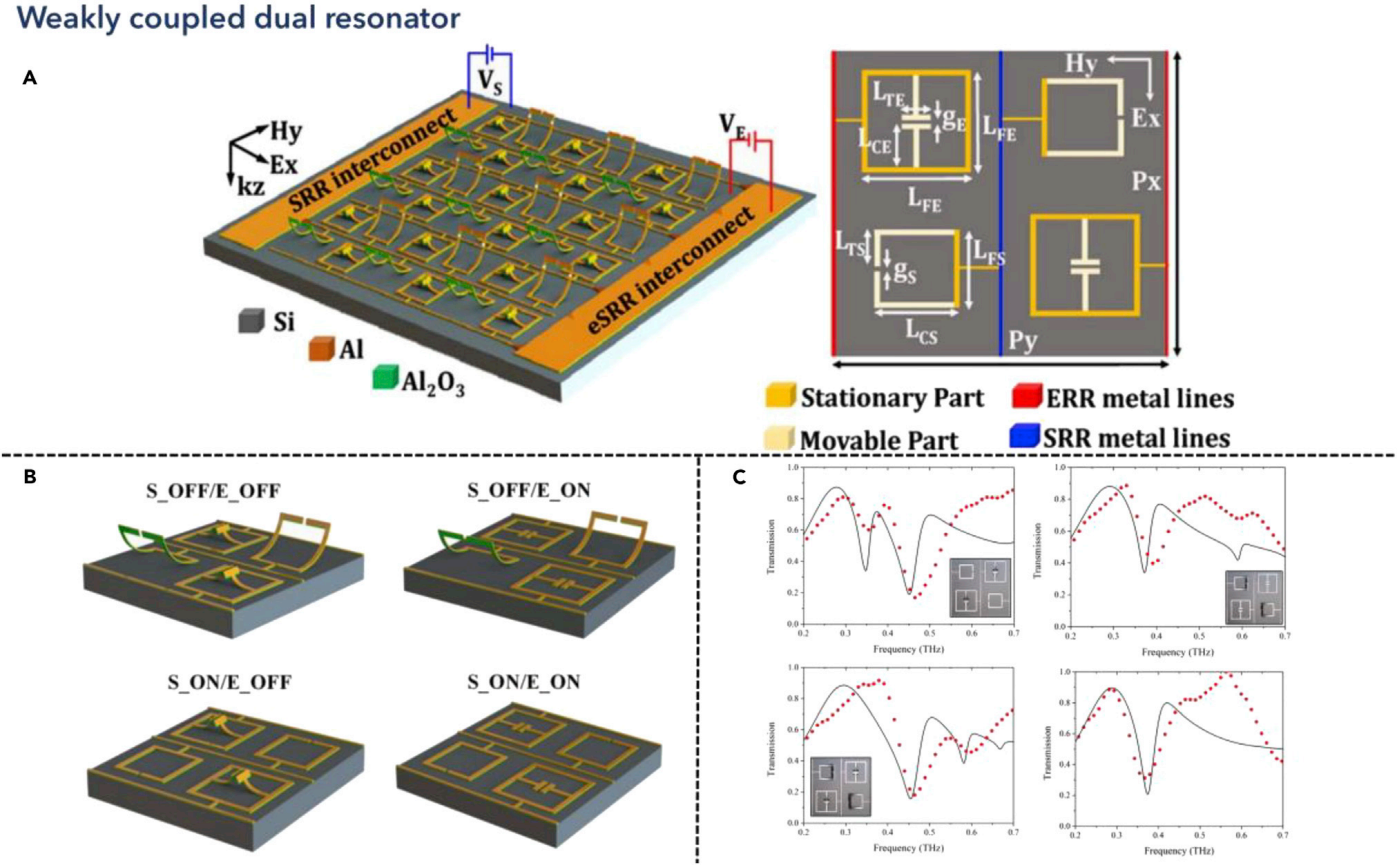
Independent dual resonance tunable metamaterials Reprinted from ref (Pitchappa et al., 2015a) with permission. Copyright@2015 Optical Society of America. (A) Schematic of interpixelated MEMS metamaterial with four various unit cell designs. (B) Four different states of the unit cells. (C) Measured results of the transmission spectra of different states.

In the weak coupling region, the coupling strength between two different modes is relatively lower, which is convenient for independent control of every single mode. One of the control methods termed programmable metamaterials has been widely utilized in the GHz spectra range, where varactor diodes could realize the tuning process (Cui et al., 2014). However, when the frequency becomes higher, one of the severe problems is to localize the control within the unit meta-atom, rather than globally control the device. Based on that, Pitchappa et al. provided possible solutions toward THz applications in 2015, where reconfigurable metamaterial for independent tuning of multiple resonances was proposed, as shown in Figure 6 (Pitchappa et al., 2015a). The metamaterial structure consisted of two parts, the cantilever-shaped beam and the SRR-shaped beam, respectively. These two shapes of the beam were staggered, where the external electric field was applied to different structures independently. Therefore, by controlling the applied voltage, the two resonances could be tuned simultaneously, as shown in Figure 6A. Apart from achieving independent control of different structures, a metacell was also proposed to realize various states control, which was made of four various structures. Thereby, by controlling the on-off states of the external bias, the cantilevers were either released or remained unmoved. Consequently, four different working states were formed by arranging the applied sequence, as depicted in Figure 6B. Furthermore, from the spectra results, we could observe independent tuning for transmission dips in the spectra, where the two resonances were activated electrically and magnetically, respectively. Besides, the working frequency range for electric and magnetic resonance was also differed, which were from 0.45 to 0.4 THz, and from 0.59 to 0.35 THz, respectively, shown in Figure 6C. Leveraging the MEMS-based THz metamaterial actuators, the structure could realize programmable control of metadevices within a small region, where the resonance magnitude and the frequency could be tuned independently, which provided multiple tuning dimensions to the system. Besides, the decoupling between electrical and magnetic resonators could be achieved using different bias control and thereby ignoring the interference between resonances.

In addition to the separated control realized electrically and magnetically, many other solutions are suitable for isolation control for dual resonances. The independent control of different electrostatic structures could realize complete control of polarization, due to the different polarization responses of the tuned structure. In 2016, Prakash et al. proposed another structure with dual resonances, which could be controlled separately (Pitchappa et al., 2016c). Moreover, the horizontal, as well as the vertically oriented structures were sensitive to different polarizations, which functioned as a switch that could complete the control of THz anisotropy, with various shapes of transmitted signals. Such design principle could also be applicable for circularly polarized light control and realization of chiroptical response (Cong et al., 2019). Besides, except for the control of the wave shape of the spectra, active tuning of a supercell with various released heights could also manipulate the bandwidth within a certain frequency range (Shih et al., 2017). Furthermore, given several control states, one could also demonstrate digital signal operation components, which functioned as logic gates (Manjappa et al., 2018b). Apart from the active tuning process leveraging a similar design as MEMS actuators, there were also many other works utilizing material properties for dynamic control of THz waves (Yang and Lin, 2021). However, the tuning of materials usually provides only a single dimension of controlling the resonance, which lacks the ability to manipulate dual resonances independently. Therefore, advanced designs that composed multiple material tuning mechanisms need to be developed in the future.

To conclude, the dual resonance THz metamaterial designs leverage the isolation between two resonances, aiming at independent control of different resonances with low coupling strength, which is suitable for multi-functional devices. Most of the designs utilize the MEMS-tunable electrostatic actuators due to their large tuning range and feasibility of realizing the function of multiple tuning. To further explore the tuning mechanisms and the tuning performance for ultrafast applications, leveraging the material properties, as well as spatiotemporal manipulation of THz waves, could be possible solutions for future tunable designs.

### Dual resonance modulators in strong coupling regions

**Figure 7:**
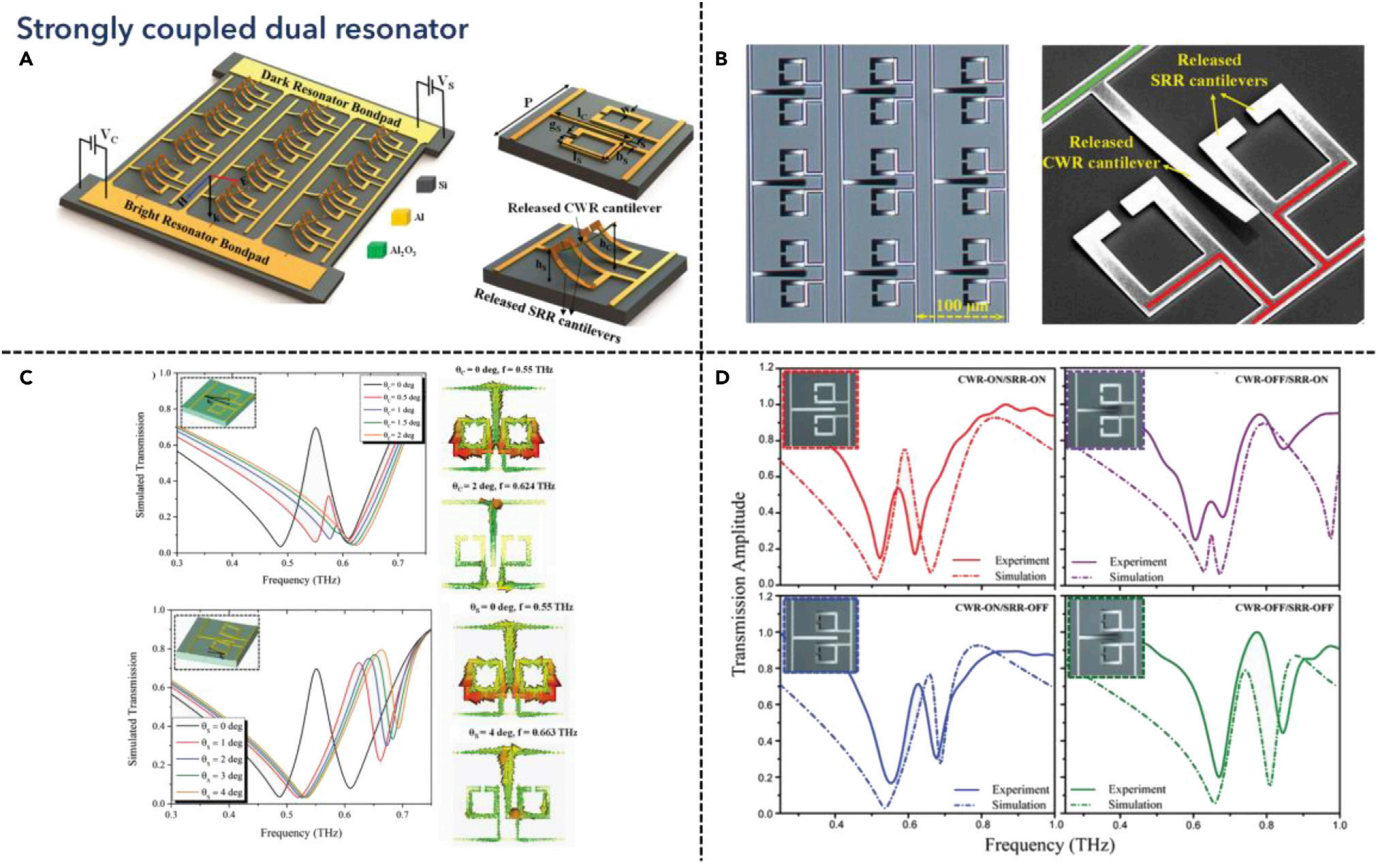
Coupled dual resonance tunable metamaterials Reprinted from ref (Pitchappa et al., 2016a) with permission. Copyright@2016 Wiley-VCH. (A) Illustration schematics of the MEMS metamaterial with independently reconfigurable released cantilever unit cells. (B) Optical microscope image and the scanning electron microscope image of the fabricated metadevice. (C) Simulated transmission spectra and the near-field image showing different cantilever structures with varied released heights. (D) Measured results showing the THz transmission response of the metamaterials off different states.

Compared with the week coupling region, the coupling between two resonances cannot be ignored for a strong coupling system, where the mode overlap influences the performance of the far-field spectra. Considering the TCMT model, the double resonances behave like a bright mode coupling with a dark mode, where the bright mode can interact with incident light and the dark mode couples to the bright mode and free space. Therefore, the dissipation rate of the two modes as well as the coupling strength between the two modes all contributes to the dual resonance spectra. In 2016, Prakash et al. proposed a new type of MEMS metamaterial resonator for THz manipulation of EIT-like resonance, as shown in Figure 7 (Pitchappa et al., 2016a). The design adapted conventional SRR as well as rod-shaped cantilever design for metamaterial resonators, made of electrostatic materials for active tuning, where the cantilever will be released when external DC bias was applied, as shown in Figure 7A. Leveraging the bright–dark coupling design principles, the rod-like resonator functioned as a bright electric dipole, which interacted with incident THz wave. While the SRRs behaved like dark mode resonators, coupling to the electric dipole mode, and could also be tuned independently, as depicted in Figure 7B. Furthermore, the spectra for two different modes were explored respectively. When the bright mode resonator was released to a certain height, the EIT-shaped peak vanished, which was attributed to the weakening of the coupling strength due to the enlargement of the in-plane gap between two resonators. Besides, when tuning the dark mode resonator, the transmitted light only behaved like a frequency shift, demonstrating that the dark mode resonator did not activate with incident THz light, as shown in Figure 7C. Consequently, by overlapping the tuning process into different states, four digital states were also demonstrated for high-speed communication systems due to the low group delay, with isolated frequency tuning and coupling strength tuning attached to different states, as shown in Figure 7D. This work further explored the possibility of leveraging MEMS technology in the active tuning of strong coupled resonant systems, where the trap mode induced coupling between bright and dark modes could be broken and modified by controlling the mechanical structures. In addition to that, similar designs were applicable in polarization-tunable devices (Pitchappa et al., 2016b), cloak (Manjappa et al., 2018a), and barcoding applications (Manjappa et al., 2018b).

Apart from reconfigurable MEMS devices, leveraging materials properties for tuning also played an important part in strong coupled resonant systems. Unlike weak coupling resonators, due to the bright–dark coupling process, the limited dimension of tuning the THz resonance is enough for dynamical tuning of materials by modifying the damping rate of the system. In 2012, Gu et al. demonstrated an active control of EIT analog in THz metamaterials, where a pair of bright mode and dark mode resonator were proposed for EIT-shaped resonance, while another photoactive layer was utilized for tuning the damping rate of dark mode resonator (Gu et al., 2012). This work illustrates the construction of the bright–dark coupling process in the THz region, followed by dynamic tuning of the coupling strength between bright mode and dark mode optically. Apart from conventional bright–dark coupling resonator designs for dual coupled resonance generation, a more efficient method was to leverage symmetry-breaking SRRs to generate high-Q Fano resonance (Cong et al., 2018). Therefore, the coupling strength can be further modified by breaking the asymmetry in the structures using material properties. Tunable materials such as phase change materials (Wang et al., 2015) and 2D materials have been successfully demonstrated due to their controllable conductivity (Manjappa and Singh, 2020), which tuned the dissipation coefficient between structures and free space. Recently, advanced materials have also become popular in the ultrafast active tuning of coupled resonances in the THz region, making one step toward the demonstration of next-generation applications. Kumar et al. proposed 2D perovskite thin films, with their exciton-mediated ultrafast free carrier relaxation, could achieve a modulation speed of around 50 GHz (Kumar et al., 2020). Dai et al. proposed high mobility 3D Dirac semimetal as a photoactive tuning layer, achieving picosecond-level tuning with a lower optical threshold, illustrating a potentially robust platform for terabit rate communications and ultrafast photodetectors (Dai et al., 2021).

In summary, strongly coupled resonance provides one more dimension for the active tuning process, which is not only compatible with MEMS technology and fabrication process but also potential in material tuning applications. Advanced development for ultrafast applications needs to be further explored for high-performance and robust commercial applications.

## Application of tunable THz metamaterial devices

### Amplitude modulation

**Figure 8:**
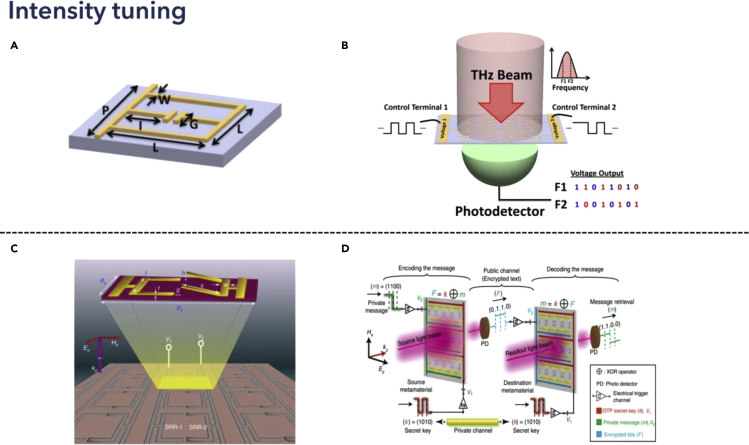
Intensity tunable THz metadevices for logic gate applications Reprinted from ref (Ho et al., 2016; Manjappa et al., 2018b) with permission. Copyright@2016 AIP Publishing, Copyright@2018 Springer Nature. (A) Schematic showing of the meta-bit structure. (B) Binary-coded digital metamaterial for the control of output intensities at specific frequencies to provide EM analogous to logic outputs. Reprinted from ref (Ho et al., 2016) with permission. Copyright@2016 AIP Publishing. (C) Schematic showing of double split-ring resonator metadevice. (D) Exclusive-OR (XOR) logic operation with MEMS Fano-metasurface and its significance in cryptographic wireless communication networks. Reprinted from ref (Manjappa et al., 2018b) with permission. Copyright@2018 Spring Nature.

When the intensity of THz spectra changes, the differential transmission or reflection coefficient could be regarded as a digital signal carrier. Modifying the intensity difference by tuning the modulation depth could effectively enable the digital states. Therefore, leveraging MEMS technology for tunable metamaterials, the reconfigurable coded metadevices could potentially function as a logic state in THz frequencies. In 2016, Ho et al. proposed digital reconfigurable binary-coded THz metamaterial, as shown in Figure 8A (Ho et al., 2016). The asymmetrical deformable cantilever design enabled independent control of each meta-atom by applying different voltages, realizing binary control of spectral output, shown in Figure 8A. Moreover, different voltage outputs could be encoded with different digital states, representing the logic outputs, shown in Figure 8B. Experimentally, the on–off states controlled the resonance dips at different frequencies at the THz spectra. Therefore, based on the intensities of the output at two frequencies, the characteristics could be analogous to NOR and AND, respectively. Applying such mechanisms, the design principles of programmable metamaterials with targeted logic gates were revealed. For more complicated logic gate designs, designing complex deformable metamaterial structures, as well as leveraging distinguishable digital states enable the multiplexing of different intensities for encoded information in THz communication applications. In 2018, Manjappa et al. developed multiple-input-output-states for logic operations at THz frequencies utilizing MEMS Fano metasurfaces, as depicted in Figure 8C (Manjappa et al., 2018b). The asymmetric SRR-shaped metasurface design could perform single resonance in the THz range from 0.4 to 0.8 THz. When two independent voltages were applied to the metamaterials, one more resonance dip appeared, which enabled the encoding process according to the intensity changes, as shown in Figure 8C. Besides, applying different voltages, the varied transmission coefficient at different states could be fully utilized for multiple states control, while the other independent controlled voltage enabled one more dimension for tuning the digital states. For the detailed encoding process, a secret key was prepared for the encoding and decoding process, which represented the sequence of applied voltages. Then, after the private messages were input to the channel, an encryption process was completed by implementing the function of the targeted logic gate with the illumination of specific THz light, where the output entered the public channel to be received. After receiving the signal, the readout of the original signal could only be completed with the same metamaterial structure as receiving port, and the secret key for translating the signal into retrieved messages, as shown in Figure 8D. Therefore, the encryption process consisted of both the metamaterial structure for the reconstruction of original THz spectra with a specific function of logic gates and the secret signal for private signal protection. This work not only revealed the feasibility of leveraging MEMS metasurfaces for THz logic gate devices but also discussed the possibility of utilizing such devices for the encryption of messages in THz communication applications. As the configurable of metamaterials, different logic gates can be easily developed (Yuan et al., 2020; Xu et al., 2021b). In addition to that, improving the modulation depth by increasing the modulation speed need to be further explored for future high bit-rates applications.

### Phase modulation

**Figure 9:**
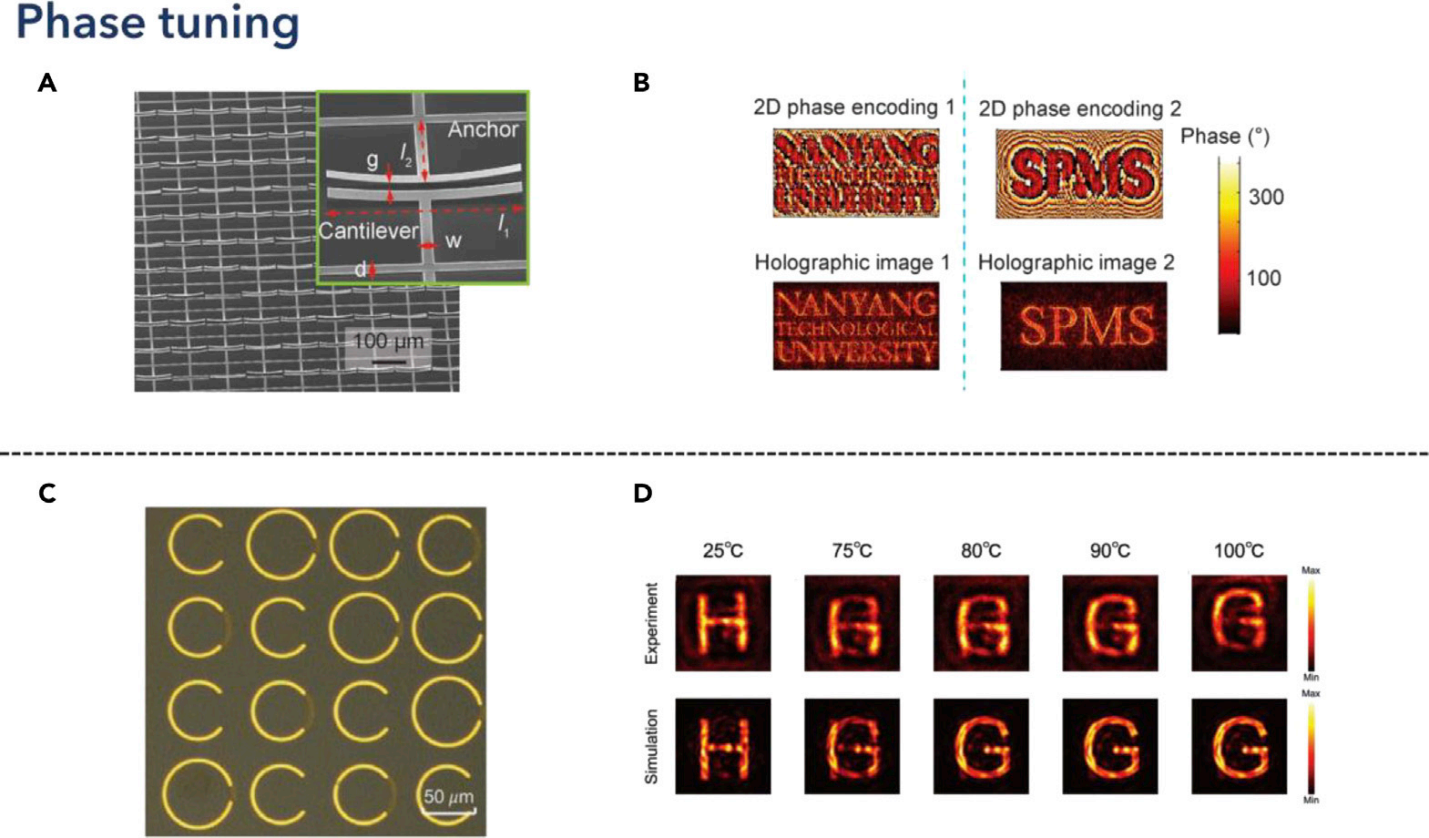
Phase tunable THz metadevices for wavefront engineering applications Reprinted from ref (Cong et al., 2017a; Liu et al., 2019b) with permission. Copyright@2017 Wiley-VCH, Copyright@2019 Wiley-VCH. (A) SEM images of the fabricated MEMS-based metadevice array. (B) The six-digit phase distribution of the two images and the corresponding holographic images displayed by encoded metadevice. Reprinted from ref (Cong et al., 2017a) with permission. Copyright@2017 Wiley-VCH. (C) Optical microscope image of the meta-hologram. (D) Measured and simulated holographic images of the dynamic meta-hologram at different temperatures. Reprinted from ref (Liu et al., 2019b) with permission. Copyright@2019 Wiley-VCH.

In resonant systems, phase gradient occurred near the resonance frequency. Modeling the principle of traditional design of the lens, artificially distributed phase profile, could enable the manipulation of phase, combined with metasurface. Functional metasurfaces could be applicable for beam steering (Cong and Singh, 2020; Tian et al., 2020; Zang et al., 2021; Zang et al., 2021), holography (Wang et al., 2017; Venkatesh et al., 2020), and focusing applications (Jia et al., 2017; Arbabi et al., 2018). In 2017, Cong et al. proposed a multi-functional MEMS device for applications in polarization control, wave deflection, and holograms, as shown in Figure 9 (Cong et al., 2017a). Deformable beam controlled the bending angle of released cantilevers when DC bias was applied, which in turn controlled the reflected phase of single unit metadevice, shown in Figure 9A. Therefore, arranging the orientation of metamaterials, the THz beam was manipulated accordingly, enabling the phase encoding for certain holographic images, where a demonstration of words “Nanyang Technological University” and “SPMS” were exhibited in Figure 9B, by applying different six-bit encoding sequences. In 2017, the phase transition mechanism was illustrated by applying the TCMT model, revealing the transition point for realizing phase gradient in holographic applications leveraging MEMS tuning mechanisms (Cong et al., 2017b). In 2019, Guo et al. proposed spatially modulated reconfigurable THz metasurface for pure phase holograms, where real-time reconstruction was demonstrated, while the modulation efficiency and imaging quality could be enhanced by increasing the pump power (Guo et al., 2019). This technology could be applicable in real-time display and optical switches for communication networks. The tunable metalens was also realized in graphene-based devices, where the electrically controlled Fermi energy could also differ the phase gradient and constructed focal plane at different distances (Liu et al., 2018). Moreover, full utilization of THz waves also enabled the designs for polarization-dependent metasurfaces, which indicated different phase transition process on each polarization state, therefore, realizing divergent holograms or deflections when rotating the polarization directions (Wang et al., 2018a), such polarization rotation can also be realized using carbon nanotubes (Baydin et al., 2021). Besides, tuning of phase profiles can also be achieved using thermally reconfigurable materials. In 2019, Liu et al. proposed thermally dependent dynamic THz metasurface using vanadium oxide, which was integrated with unit resonator structures, as shown in Figure 9C (Liu et al., 2019b). Therefore, when the temperature increased, the vanadium oxide would transform from the insulator phase to the metallic phase, thus changing the conductivity and influencing the transmitted amplitude and phase in the far-field spectra. Observing from the holographic image plane, the presented letter “H” would gradually turn into “G” when the temperature changed, as shown in Figure 9D. Similar temperature-dependent wavefront manipulation using “C”-shaped metasurface could also be leveraged for focusing applications (Wang et al., 2019a). Recently, Cong et al. proposed a new type of metasurface leveraging a high-Q dielectric resonator, where a phase transition point was observed when the dipole mode changed, which occurred when the light power reached a critical value (Cong and Singh, 2020). This work revealed ultrafast reconfigurable beam steering of THz waves leveraging dielectric high-Q resonators, opening new avenues to overcome the fundamental limitation of spatial metamaterials in 6G wireless communication applications.

## Conclusion and outlook

THz modulation devices leveraging metamaterial designs have grown in scientific and technological relevance from a physical principle to practical applications. Manipulation of THz waves shows significant advances in functional reconfigurable metamaterial devices, which enable the dynamic control of THz signals in wireless and on-chip communication, chemical sensing, and wavelength-selective radiation detection. In tunable metamaterial devices, various deformable MEMS actuators, such as electrostatic actuators, thermal actuators, etc., as well as microfluidic channels and conductive-sensitive materials, have been used to modulate metamaterial resonant systems in the THz region. Among these modulators, frequency modulation and amplitude modulation are two of the most common methods in single resonance systems. Besides, multi-resonance systems enable the controlling of coupling strength between resonances, which brings advanced applications in optical switch, logic gate, holography, beam deflection, and so on.

**Figure 10:**
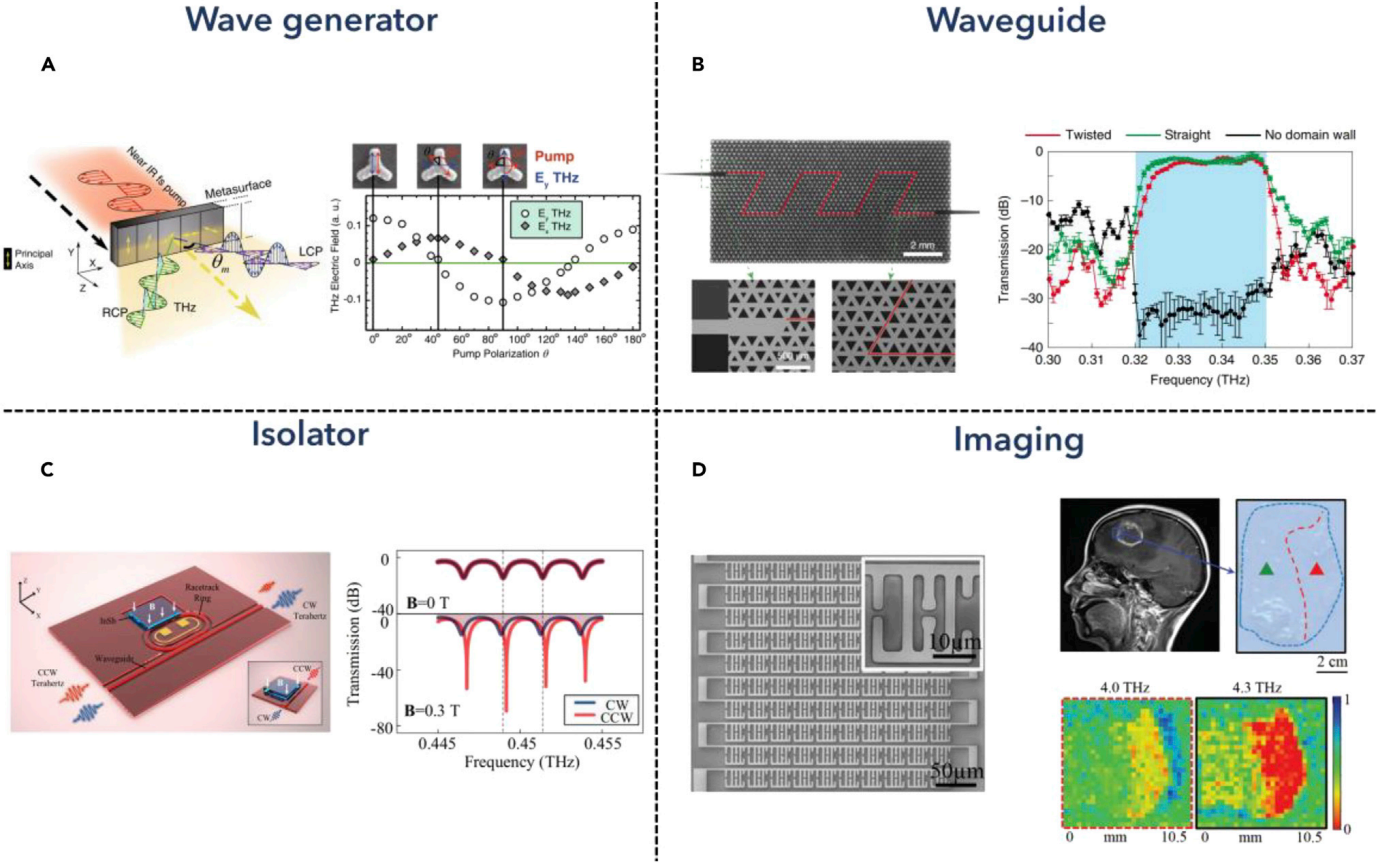
Outlook and perspective of future THz-tunable photonic devices Reprinted from ref (McDonnell et al., 2021; Yang et al., 2020; Yuan et al., 2021; Zhou et al., 2018) with permission. Copyright@2021 Springer Nature, Copyright@2020 Springer Nature, Copyright@2021 Springer Nature, Copyright@2018 Wiley-VCH. (A) Symmetry selection rules for generating THz pulses from a C3 meta-atom, and corresponding frequency spectrum for the generated THz pulse. Reprinted from ref (McDonnell et al., 2021) with permission. Copyright@2021 Spring Nature. (B) Topological photonics for on-chip wave transmission with its domain wall, and the measured transmission curves. Reprinted from ref (Yang et al., 2020) with permission. Copyright@2020 Spring Nature. (C) On-chip THz isolator structure, with the transmission properties of the chip in reciprocal and nonreciprocal states. Reprinted from ref (Yuan et al., 2021) with permission. Copyright@2021 Spring Nature. (D) A portion of the metamaterial focal plane arrays detector, with the reconstructed image between normal tissue and brain metastasis. Reprinted from ref (Zhou et al., 2018) with permission. Copyright@2018 Spring Nature.

In the future, stepping into 6G wireless communication, advanced THz integrated circuits needs to be developed for commercial applications. The lack of high-efficiency THz sources, high-sensitive detectors, and other functional devices are the key factors that block the development of the technology. Therefore, advanced designs of THz photonics devices need to be put forward for future applications. Recently, McDonnell et al. proposed a function THz emitter based on Pancharatnam-Berry phase nonlinear metasurface, which allowed control over polarization and phase of the THz wave packet, enabling circular polarization state change in the time domain, as shown in Figure 10A (McDonnell et al., 2021). Such functional THz wave generator containing polarization tunability could be utilized in wireless communication with its intrinsic polarization modulation, or enantiomer sensing due to its circular dichroism response. For on-chip communication systems, the propagation of THz waves is also one of the most significant components to be explored. In 2020, Yang et al. proposed a THz topological photonic crystal waveguide for THz transport application and demonstrated uncompressed 4K high-definition video transmission (Yang et al., 2020). The topological valley kink states functioned as excellent information carriers for THz communication with a high transfer rate and low bit error rate, where a domain wall enabled high-efficiency transmission of THz waves, as shown in Figure 10B. Moreover, the all-silicon chip design enabled CMOS-compatible fabrication processes, which had the potential for commercial applications in the future. Apart from waveguide designs, recently, tremendous on-chip THz devices have also been explored and demonstrated (Wu et al., 2019; Kokkoniemi et al., 2020). Recently, Yuan et al. proposed an on-chip THz isolator design, which leveraged the magneto-optical effect of a nonreciprocal resonator to isolate clockwise and counter-clockwise THz waves by varying the magnetic field intensity with an isolation ratio of 52 dB, as shown in Figure 10C (Yuan et al., 2021). Furthermore, to improve the functionality of the on-chip devices, electrically driven tunability of the isolator was also demonstrated thermally, where the central frequency shifted as the external current was applied. In addition to THz communication devices, necessary attention could be also paid to functional THz devices for other applications. In 2018, Zhou et al. proposed a multispectral metamaterials array for multicolor application, as shown in Figure 10D (Zhou et al., 2018). The optimized metamaterial design consisted of four by four pixels with different detection sensitivity. Therefore, a demonstration showed that at 4.0 THz and 4.3 THz, the boundary between metastatic brain tumor and normal brain tissue on the MRI image of a patient could be distinguished with different transmitted intensity. This non-invasive, subwavelength imaging system could be further improved with higher resolution and faster response rate, broadening the applications for intriguing prospects, such as biological systems (Lee et al., 2020) (Zhan et al., 2021).

In conclusion, with the rapid development of critical science and technology in recent years, metamaterial-based THz photonics have been developed toward a standard and robust platform to achieve basic tunability for more functional applications. On the one hand, higher tunability, faster response rate, and lower power consumption are the challenges that remain for more commercial applications (Li et al., 2020a) (Manjappa and Singh, 2020; Agarwal et al., 2021; Dong et al., 2021; Pitchappa et al., 2021b). On the other hand, systematical work that required high integration and feasible software platform is also current blocks to be overcome (Wu et al., 2019; Wan et al., 2021). All in all, the combination of high-performance THz-tunable devices, together with other functional devices could enable powerful applications in healthcare, 6G wireless communication, and IoT regions.
